# Costimulatory Function of Cd58/Cd2 Interaction in Adaptive Humoral Immunity in a Zebrafish Model

**DOI:** 10.3389/fimmu.2018.01204

**Published:** 2018-05-31

**Authors:** Tong Shao, Wei Shi, Jia-yu Zheng, Xiao-xiao Xu, Ai-fu Lin, Li-xin Xiang, Jian-zhong Shao

**Affiliations:** ^1^College of Life Sciences, Key Laboratory for Cell and Gene Engineering of Zhejiang Province, Zhejiang University, Hangzhou, China; ^2^Laboratory for Marine Biology and Biotechnology, Qingdao National Laboratory for Marine Science and Technology, Qingdao, China

**Keywords:** *cd58*, *cd2*, costimulatory signals, Cd4^+^ T cells, adaptive humoral immunity, zebrafish

## Abstract

CD58 and CD2 have long been known as a pair of reciprocal adhesion molecules involved in the immune modulations of CD8^+^ T and NK-mediated cellular immunity in humans and several other mammals. However, the functional roles of CD58 and CD2 in CD4^+^ T-mediated adaptive humoral immunity remain poorly defined. Moreover, the current functional observations of CD58 and CD2 were mainly acquired from *in vitro* assays, and *in vivo* investigation is greatly limited due to the absence of a *Cd58* homology in murine models. In this study, we identified *cd58* and *cd2* homologs from the model species zebrafish (*Danio rerio*). These two molecules share conserved structural features to their mammalian counterparts. Functionally, *cd58* and *cd2* were significantly upregulated on antigen-presenting cells and Cd4^+^ T cells upon antigen stimulation. Blockade or knockdown of Cd58 and Cd2 dramatically impaired the activation of antigen-specific Cd4^+^ T and mIgM^+^ B cells, followed by the inhibition of antibody production and host defense against bacterial infections. These results indicate that CD58/CD2 interaction was required for the full activation of CD4^+^ T-mediated adaptive humoral immunity. The interaction of Cd58 with Cd2 was confirmed by co-immunoprecipitation and functional competitive assays by introducing a soluble Cd2 protein. This study highlights a new costimulatory mechanism underlying the regulatory network of adaptive immunity and makes zebrafish an attractive model organism for the investigation of CD58/CD2-mediated immunology and disorders. It also provides a cross-species understanding of the evolutionary history of costimulatory signals from fish to mammals as a whole.

## Introduction

CD58, also known as lymphocyte function-associated antigen-3, was first identified from humans (*Homo sapiens*) as an adhesion molecule in the 1980s (1, 2). It is a heavily glycosylated protein whose extracellular region contains a single V-set and a C2-set Ig superfamily (IgSF) domain (3–5). CD58 was expressed on the surface of human hemopoietic and non-hemopoietic lineages, including dendritic cells, macrophages, endothelial cells, and erythrocytes in a transmembrane and glycosylphosphatidylinositol (GPI)-anchored form (6–11). CD58 was also identified from several other mammals, including porcine (*Sus scrofa*) and sheep (*Ovis aries*) (12, 13). Unfortunately, a CD58 homolog has still not been identified in murine models; therefore, functional investigations on CD58 are greatly limited (14). Several previous studies in humans have shown the involvement of CD58 in T-cell cytokine production, T-cell responsiveness to IL-12, induction of TNF-α and IL-1β from monocytes, and IgE production by B cells (15–17). Blockade of CD58 by anti-CD58 monoclonal antibodies and a CD58-Ig fusion protein can reduce inflammatory responses and diminish the recognition and cytolysis of target cells by cytotoxic T lymphocytes and NK cells (2, 18–20). These findings suggest that CD58 plays important roles in both innate and adaptive immunities, with a particularly regulatory role at the effector and target cell levels (2, 9).

In most cases, CD58 exerts its functions through the interaction with its receptor CD2 molecule (15, 21). CD2 has been called lymphocyte function-associated antigen-2. It is also a member of the immunoglobulin superfamily, which is expressed on the surface of almost all mature peripheral T cells, thymocytes, NK cells, and thymic B cells (3, 22, 23). The interaction of CD58 with CD2 has been found to be essential for the activation of cellular immunity, such as CD8^+^ cytotoxic T lymphocytes and NK cell-mediated cytotoxic reactions (3, 24, 25). However, functional characterization of CD58 with CD2 in CD4^+^ T-cell-mediated adaptive humoral immunity remains limited. Also, previous studies on CD58 and CD2 have tended to be confined to humans *in vitro*. The precise functional roles of these two reciprocal molecules *in vivo* still need to be elucidated, which largely depends on the establishment of a model organism to compensate for the limitation of humans.

In this study, we characterized *cd58* (si:dkey-11f4.14) and *cd2* (si:ch211-132g1.1) homologs from a zebrafish (*Danio rerio*) model and uncovered their costimulatory functions in the activation of adaptive humoral immunity. To the best of our knowledge, this study is the first to prove that functional CD58 and CD2 homologs exist in a lower vertebrate and to highlight a novel costimulatory mechanism underlying adaptive immunity. The findings contribute to the current knowledge on CD58/CD2-mediated immunity and provide a cross-species understanding of the evolutionary history of the costimulatory systems from fish to mammals. Furthermore, this study showed again the advantage of zebrafish as an attractive model system in uncovering the mechanisms underlying innate and adaptive immunity as previously suggested (26). Given the absence of CD58 homolog in mice, the mainstay of immunological research animal model, zebrafish, is expected to be a compensation for the lack of murine models. Moreover, CD58 and CD2 have attracted attention because of their involvement in various diseases, such as rheumatic arthritis, multiple sclerosis, colorectal cancer, and diffuse large B cell lymphoma (27–30). This study would also provide an opportunity to develop a zebrafish model for the clinical investigations or medical applications of CD58/CD2-based therapies.

## Materials and Methods

### Experimental Fish

Wild-type AB zebrafish (*Danio rerio*) of both sexes, 1-year old and approximately 0.5–1.0 g in weight, were offspring of a single parent pair after five generations of partial inbreeding in our laboratory as previously described (31–33). All fish were maintained in circulating water at 28°C under standard conditions and held for at least 2 weeks before use in experiments for evaluation of overall health. All animal work in this paper was conducted according to relevant national and international guidelines. All animal care and experimental procedures were approved by the Committee on Animal Care and Use and the Committee on the Ethic of Animal Experiments of Zhejiang University.

### Molecular Cloning

Cd58 and Cd2 amino acid sequences were obtained through BLASTp in NCBI and the ORF of *cd58* and *cd2* were searched by the target sequences. PCR were performed with the cDNA library acquired from spleen and head kidney and the specific primers (shown in Table S1 in Supplementary Material) of *cd58* and *cd2*, respectively. Afterward, the target DNA fragments were obtained by the agarose gel electrophoresis with the Gel Extract Kit (Omega). After adding adenine, the target DNA fragments were equipped to the GEM T Easy vector using T4 DNA ligase (Promega). The plasmids were transformed into competent *Escherichia coli* DH5α (Takara). The positive plasmid DNA was purified following the Miniprep protocol (OMEGA) and sequenced on an ABI 3730XL Sequencer (Invitrogen).

### Bioinformatics Analysis

Full-length *cd58* and *cd2* cDNAs were assembled using the CAP3 Sequence Assembly Program. Genome assemblies and locations were retrieved from the University of California at Santa Cruz genome bioinformatics website and map viewer in the NCBI. By comparing *cd58* and *cd2* cDNAs with genome sequences, gene organizations (intron/exon boundaries) were elucidated and figures were drawn with GeneMapper 2.5. Using the ClustalX program (version 3.0), MEGA 4.1 software and the BLASTp algorithm, multiple alignments, and phylogenetic trees were generated (34, 35). The signal peptide, transmembrane domain, and potential functional motifs were predicted using SignalP 4.1 Server, TMHMM Server 2.0, and PROSITE (36–38). N-linked glycosylation sites were predicted using NetNGlyc 1.0 Server (39). Secondary and 3D-structures were analyzed using SMART, SWISS-MODEL, and I-TASSER (40–42). The crystal structures of *Hs*CD58 (PDB 1CCZ) and *Hs*CD2 (PDB 1HNF) were used as templates to build the models, respectively.

### Plasmid Constructs and Recombinant Proteins

The coding sequences for the ORF or the extracellular domain of *cd58* and *cd2* were amplified through RT-PCR by using primers (shown in Table S1 in Supplementary Material) containing an EcoRI site added to the 5′ end and an XhoI site added to the 3′ end. The PCR products were digested and ligated into pEGFP-N1 (Clontech) or pcDNA6/myc-His©B (Invitrogen) to construct eukaryotic expression vectors (pEGFP-*cd58*, pEGFP-*cd2*, and pcDNA6-*cd58*) with enhanced GFP-tag or myc-tag and into pMalc2e to construct prokaryotic expression vectors (pMalc2e-*cd2*) with MBP-tag (43). For eukaryotic expression of Cd58 protein, the plasmid DNA was transformed into HEK293T cells. For prokaryotic expression of Cd2 protein, the pMalc2e-*cd2* was transformed into *E*. *coli* Rosetta (DE3) pLysS. Positive colonies were inoculated into Luria–Bertani medium containing kanamycin (50 µg/mL) and the protein expression was induced by isopropyl-β-d-thio-galactoside (1 mM/mL) as previously described (31). The recombinant proteins were detected *via* SDS-PAGE and purified through Amylose resin affinity chromatography in accordance with the manufacturer’s manual (NEB, pMAL system).

### Preparation of Polyclonal Antibodies (Abs)

Antibodies against Cd58 and Cd2 were produced by epitope-peptide or recombinant protein immunized approach. Briefly, the epitope sequences on Cd58 surface were predicted by ABCPred, BepiPred, MAPPP, and IEDB online softwares and confirmed by 3D structure modeling through utilizing SWISS-MODEL program. The amino acid sequences were chemically synthesized, purified through HPLC, and coupled to ovalbumin (OVA) at a ratio of 10 mg:10 mg (carrier/peptide) as previously described (44). New Zealand white rabbits (~1.5 kg) and Balb/c mice (~25 g) were immunized with OVA-coupled peptides (1 mg for rabbits) or recombinant Cd2 protein (10 µg for mouse) in CFA initially and then in IFA four times thereafter at biweekly intervals. One week after the final immunization, antiserum samples were collected from the animals, and the Abs were affinity-purified into IgG isotype by using a protein A agarose column (Qiagen) and a membrane-based Ag-absorbent protocol as previously described (32, 44, 45). The Abs titers were determined by ELISA, and the specificity was characterized by Western blot. The Abs against zebrafish MHC class II (Mhc-ii), mIgM, Cd4, Cd80/86, Cd83, Tcr-α or Tcr-β, Cd40 and Cd154, including mouse anti-Mhc-ii, mouse anti-mIgM, mouse anti-Cd80/86, mouse anti-Cd83, mouse anti-Cd4, mouse anti-Cd40, rabbit anti-Tcr-α, rabbit anti-Tcr-β, rabbit anti-Cd4, rabbit anti-Cd40, rabbit anti-mIgM, and rabbit anti-Cd154 were produced in our previous studies (31, 32, 44–46).

### Generation of Small Interfering RNA (siRNA) Encoding Lentivirus (LV)

Short hairpin RNA (shRNA) containing the siRNAs targeting to *cd58* or the scrambled siRNA was designed as previously described (shown in Table S1 in Supplementary Material) (31, 32). The shRNA was constructed into pSUPER vector (pSUPER.retro.puro; Oligoengine, Seattle, WA, USA) downstream of the H1 promoter. The reconstructed plasmids were cotransfected into HEK293T cells with pcDNA6-*cd58*. The U6 promoter cassette in lentiviral vector pLB was replaced by H1-siRNA cassette excised from the highly effective siRNA construct screened to produce pLB-*cd58* lentiviral vector. The constructed pLB-*cd58* was cotransfected with pCMV-dR8.2 dvpr and pCMV-VSVG packaging vectors into HEK293T cells in a proportion of 10:7:3 by using polyethylenimine. The lentiviral supernatant was concentrated *via* ultracentrifugation in 4°C, at 25,000 *g*, for 90 min, and the viral titers were detected through flow cytometry (FCM) analysis of EGFP expression in HEK293T cells. A fluorescent microscope (Zeiss Axiovert 40 CFL; Carl Zeiss, Oberkochen, Germany) was used to examine infected cells. The silencing efficacy of the LV (*cd58*siRNA-LV) was determined in HEK293T cells transfected with pcDNA6-*cd58* and in peripheral blood, spleen, and kidney leukocytes by real-time PCR or FCM analysis after the cells were infected with *cd58*siRNA-LV (2 × 10^5^ TU/mL) for 72 h, and the zebrafish were intraperitoneal (i.p.) injected with *cd58*siRNA-LV (2 × 10^5^ TU/fish) once every 24 h for three times (31, 32).

### Subcellular Localization Analysis

HEK293T cells (5 × 10^5^/mL) were seeded into 12-well plates (Corning Inc.) and then cultured in DMEM (Gibco, USA) supplemented with 10% FBS (Gibco, USA) at 37°C in 5% CO_2_ to allow growth until 70–80% confluence was reached. Then, 0.75 µg pEGFPN1-*cd58* or pEGFPN1-*cd2* plasmid DNA combined with FuGENE^®^ HD Transfection Reagent (Roche, 3 μL/well) were transiently cotransfected into HEK293T cells according to the manufacturer’s instructions. At 48 h post-transfection, 2% paraformaldehyde was used to fix the cells for 10 min, and then the cells were stained with 10 µM DiI (Beyotime) and 100 ng/mL DAPI (Sigma) at 37°C for 5 min. Fluorescence images of Cd58 and Cd2 were obtained using a two-photon laser scanning confocal microscope (Zeiss LSM710, Germany) with 630× magnification.

### Magnetic-Activated Cell Sorting for APCs and Cd4^+^ T Cells

Zebrafish were i.p. injected with 10 µg of keyhole limpet hemocyanin (KLH, Sigma-Aldrich) and 10 ng of lipopolysaccharide (LPS, Sigma-Aldrich) from *E. coli* serotypes O55:B5 or 2 × 10^5^ CFU *Aeromonas hydrophila*. Leukocytes were collected from the spleen, head kidney, and peripheral blood through Ficoll-Hypaque (1.080 g/mL) density-gradient centrifugation as previously described (31–33, 44). The cells were blocked in D-Hank’s buffer with 2% BSA for 2 h, and then the leukocytes (about 1 × 10^8^/mL) were incubated in 2% BSA with mouse anti-Cd4 Ab or mouse anti-Mhc-ii Ab for 1 h at 4°C. After the incubation, the cells were gently washed thrice with D-Hank’s buffer, incubated with anti-mouse IgG magnetic beads (Thermo Scientific) for 15 min at 4°C, and then applied to a magnetic separator to separate the target cells which were Mhc-ii^+^ or Cd4^+^. The Mhc-ii^+^ and Cd4^+^ cells were cultured in L-15 medium (Gibco) containing 10% FBS (Gibco), 100 U/mL penicillin, and 100 µg/mL streptomycin at 28°C overnight to detach the magnetic beads (32, 44).

### Real-Time PCR

Total RNA was extracted from tissues and leukocytes from the spleen, kidney, and peripheral blood. The transcripts of *cd58* and *cd2* were analyzed *via* quantitative real-time PCR with primers shown in Table S1 in Supplementary Material on a Mastercycler Ep Realplex instrument (Eppendorf). The efficiency of each primer was determined by making serial dilutions of pooled cDNA and calculating a linear regression based on the CT data points as previously described (47). The primers with efficiencies varied between 95 and 105% were used in the study. All PCR experiments were performed in a total volume of 10 µL by using a SYBR Premix Ex Taq kit (Takara Bio). The PCR program was as follows: (1) 94°C for 2 min; (2) 40 cycles of denaturation at 94°C for 20 s, annealing at 55°C to 65°C for 20 s, and extension at 72°C for 20 s; (3) melting curve analysis at 95°C for 15 s, 60°C for 15 s, 60°C up to 95°C for 20 min, and 95°C for 15 s; and (4) cooling at 40°C for 30 s. Using the 2^−ΔΔCT^ method with β-actin as reference gene, the relative expression levels were calculated. In all cases, the sample was run in triplicate parallel reactions, and each experiment was repeated at least three times independently.

### Flow Cytometric Analysis

Cells under examination were blocked with 2% BSA for 2 h at 4°C and then incubated with the defined primary Abs for 1 h at 4°C. Nonspecific rabbit or mouse IgG was served as the negative control. After washing twice with D-Hank’s buffer, the cells were incubated with secondary Abs (PE conjugated goat anti-mouse and FITC conjugated goat anti-rabbit) for 1 h at 4°C, and the fluorescence signals were determined using the flow cytometer (BD FACSCalibur). At least 10,000 cells were collected from the lymphocyte gate for analysis (31, 32, 44). Cell Quest software (BD Biosciences) and ModFit LT software were used for FCM analyses and T cell proliferation assays, respectively (31, 32, 44).

### Immunofluorescence Staining

Co-localizations of Cd58 with Mhc-ii, Cd80/86, or Cd83 and Cd2 with Cd4, Tcr-α, or Tcr-β were detected *via* immunofluorescence staining, respectively. Leukocytes acquired from Ficoll-Hypaque (1.080 g/mL) centrifugation were separated from zebrafish stimulated by PBS or 10 µg of KLH (Sigma-Aldrich) and 10 ng of LPS (Sigma-Aldrich). The leukocytes were fixed with 2% paraformaldehyde at 25°C for 10 min, blocked with 2% BSA, and then incubated with primary Abs (rabbit anti-Cd58 along with mouse anti-Mhc-ii, mouse anti-Cd80/86, or mouse anti-Cd83, and mouse anti-Cd2 along with rabbit anti-Cd4, rabbit anti-Tcr-α, or rabbit anti-Tcr-β) at 4°C for 1 h. After being washed with 0.9% PBS, the cells were incubated with secondary Abs (PE-conjugated anti-mouse Abs and FITC-conjugated anti-rabbit Abs; Thermo Scientific) according to the manufacturer’s instructions (31, 32, 44). Then, the cells were incubated with 0.1% DAPI (Invitrogen) at 25°C for 5 min to stain the nucleus. Nonspecific rabbit or mouse IgG was served as the negative control. Imaging was performed under a two-photon laser-scanning microscope (Zeiss LSM710, Germany) with 630× magnification.

### Induced Expression of Cd58 on APCs and Cd2 on Cd4^+^ T Cells

Induced expression of *cd58* on APCs and *cd2* on Cd4^+^ T cells in response to antigen stimulation was examined *in vivo* and/or *in vitro* as previously described (32). For *in vivo* assay, fish were i.p. injected with KLH (10 µg/fish), LPS (10 ng/fish), KLH (10 µg/fish) plus LPS (10 ng/fish), or *A. hydrophila* (2 × 10^5^ CFU/fish). After 72 h, the double-positive cells in leukocytes from the spleen, kidney, and peripheral blood were labeled with primary Abs (mouse anti-Mhc-ii along with rabbit anti-Cd58; and mouse anti-Cd2 along with rabbit anti-Cd4) and secondary Abs (PE-conjugated anti-mouse Abs and FITC-conjugated anti-rabbit Abs, Thermo Scientific), and then analyzed *via* FCM as described above. For *in vitro* assay, primary Mhc-ii^+^ APCs cells were sorted from untreated fish and then stimulated with KLH in different combinations (10 µg KLH, 100 ng LPS, 10 µg KLH plus 100 ng LPS, or *A. hydrophila*, 2 × 10^6^ CFU/mL). After 8 h of incubation, the cells were harvested, and the expression levels of Cd58 were examined by FCM. The expression of *cd58* and *cd2* was also examined by real-time PCR with primers shown in Table S1 in Supplementary Material.

### *In Vitro* Assay for Cd58 and Cd2 on Cd4^+^ T Cell Activation

Fish were i.p. injected with 10 µg of KLH plus 10 ng of LPS or *A*. *hydrophila* (2 × 10^5^ CFU/fish) 5 days before sacrificed. Cd4^+^ T cells were magnetically sorted from the spleen, kidney, and peripheral blood leukocytes (PBLs) with anti-Cd4 Ab; stained with 5 µM CFSE (Beyotime); and then terminated by adding 10% FBS as previously described (31, 32, 44). Mhc-ii^+^ APCs were also magnetically sorted and beforehand stimulated with KLH (100 µg/mL) plus 100 ng/mL of LPS or *A*. *hydrophila* (2 × 10^5^ CFU/mL) for 8 h. The APCs were cocultured with Cd4^+^ T cells for 72 h, during which rabbit anti-Cd58 or mouse anti-Cd2 Ab (5 µg/mL) and nonrelated rabbit IgG (5 µg/mL), were added into the cultures every 24 h for 3 days. The proliferation and activation of antigen-specific Cd4^+^ T cells (Cd4^+^Cd154^+^ T_KLH/_*_A.h_*) were examined *via* FCM, and the expression of *cd154, lck* (tyrosine-protein kinase), *il-4/13a, il-4/13b, il-2*, and *ifn-γ* was detected using real-time PCR (primers shown in Table S1 in Supplementary Material) (48).

### *In Vivo* Assay for Cd58 on Cd4^+^ T Cell Activation

*In vivo* knockdown and blockade assays were performed to evaluate the effect of Cd58 on Cd4^+^ T cell activation. For the knockdown assay, fish were i.p. injected thrice with siRNA-encoding LV (2 × 10^5^ TU/fish) at a 24-h interval (31, 32). At the last administration, the samples were coinjected with 10 µg of KLH plus 10 ng of LPS or 2 × 10^5^ CFU *A. hydrophila*. Scrambled siRNA-encoding LV was administered as a negative control (31, 32). For the blockade assay, fish were i.p. injected with 10 µg of KLH plus 10 ng of LPS or 2 × 10^5^ CFU *A. hydrophila* and then administered thrice with anti-Cd58 Ab (10 µg/fish) at a 24-h interval. Nonrelated rabbit IgG was injected (10 µg/fish) as an isotype control. The proliferation and activation of antigen-specific Cd4^+^ T cells (Cd4^+^Cd154^+^ T_KLH/_*_A.h_*) were examined *via* FCM, and the expression of *cd154* and *lck* in PBLs and head kidney lymphocytes (HKLs) was detected using real-time PCR.

### Effect of Cd58 on B Cell Activation and IgM Production

For the B cell activation assay, fish were i.p. injected with 10 µg of KLH and then administered thrice with anti-Cd58 Ab (10 µg/fish) at a 24-h interval. After 4 days stimulation with KLH, leukocytes from the spleen, kidney, and peripheral blood were collected, and the proliferation and activation of B cells were assessed as the increase of IgM^+^Cd40^+^ cells *via* FCM with mouse derived anti-mIgM Ab and rabbit derived anti-Cd40 Ab (31, 46). For the IgM production assay, fish were i.p. immunized with 10 µg of KLH, administered thrice with anti-Cd58 Ab or anti-Cd2 at a 24-h interval, and then further immunized on the fifth and eighth day. Serum samples were collected at 35 days after the first immunization, and the level of IgM against KLH was measured using ELISA as previously reported (31, 44). Briefly, KLH (5 µg/mL) was used to coat 96-well ELISA plates overnight at 4°C. Then, coated 96-well ELISA plates were blocked with 2% BSA for 1 h at 37°C and washed with PBST (PBS with 0.05% Tween-20). After that, the wells were loaded with serially diluted zebrafish serum samples at 37°C. 2 h of incubation later, the plates were washed thrice with PBST and incubated with rabbit anti-IgM Ab for 1 h at 37°C. Afterward, the plates were washed, and the HRP-conjugated goat anti-rabbit-IgG Ab was added. Color was developed using tetramethylbenzidine and stopped with 2 mol/L H_2_SO_4_, and then measured at 450 nm on a Synergy H1 Hybrid Reader (BioTek Instruments). Ab titer is defined as the highest dilution of serum at which the A450 ratio (A450 of post-immunization sera/A450 of pre-immunization sera) is greater than 2.1 (31, 46).

### Immunoprotection Assay

Immunoprotection assay was performed to further evaluate the role of Cd58 in adaptive humoral immunity with the immunized groups and unimmunized control group. One of the immunized group was i.p. immunized with a bacterial vaccine (2 × 10^5^ CFU) derived from 0.5% formaldehyde-inactivated *A. hydrophila*, a pathogen of infectious sepsis in fish (46, 49, 50). The other immunized group was also immunized with the same *A. hydrophila* vaccine in the same dosage except the administration of anti-Cd58 (10 µg/fish) as described above. 35 days later, all the groups were challenged with living *A. hydrophila* (2 × 10^5^ CFU/fish). The mortality of each group was recorded, and the statistics of survival were analyzed (31, 46).

### Functional Evaluation of the Association Between Cd58 and Cd2

To investigate whether the role of Cd58 in the activation of adaptive humoral immunity is associated with Cd2, a functional evaluation was performed by the examination of the regulatory role of Cd2 in Cd4^+^ T_KLH_/Cd4^+^ T*_A_*_._*_h_* activation and Cd4^+^ T_KLH_/Cd4^+^ T*_A_*_._*_h_*-initiated B cell activation. For this procedure, fish were challenged with 10 µg of KLH plus 10 ng of LPS or 2 × 10^5^ CFU *A. hydrophila* and then administered thrice (at a 24-h interval) with the soluble Cd2 protein (sCd2) at different concentrations (1, 5, and 10 µg). MBP-tag protein (10 µg) was i.p. injected as a negative control. The leukocytes were isolated from the spleen, kidney, and peripheral blood tissues 3 days after antigen stimulation. The proliferation and activation of Cd4^+^ T and B cells were assessed by the increase of Cd4^+^Cd154^+^ T and IgM^+^Cd40^+^ B cells *via* FCM, and the upregulation of *cd154* and *lck* (for Cd4^+^ T cells) *via* real-time PCR as described above.

### Co-Immunoprecipitation (CoIP) Assay for Association Between Cd58 and Cd2

Association between Cd58 *and* Cd2 was examined *via* CoIP and FCM. For CoIP procedure, HEK293T cells were co-transfected with pcDNA6-*cd58* fused with myc-tag and pEGFP-*cd2* fused with GFP-tag. At 48 h post-transfection, the supernatant was discarded, and the cells were washed with serum-free buffer, then treated by 450 µL of lysis buffer (1% Triton X-100, 150 mM NaCl, 1 mM EDTA, 0.1% SDS, 1% sodium deoxycholate, 50 mM Tris–HCl, pH 7.4) with protease inhibitor cocktail (Roche) for 30 min at 4°C. The cells lysis products were then centrifuged for 10 min at 12,000 *g* to collect the supernatant, followed by incubating with mouse anti-GFP Ab (Abcam) at 4°C overnight. Next day, the supernatant was added with up to 50 µL of protein G-agarose bead (Sigma-Aldrich), shook for 4 h at 4°C, and washed thrice with cold PBS. The precipitants were denatured in loading buffer for analysis by Western blot using 12% SDS-PAGE. For FCM, pcDNA6-*cd58* were transfected into HEK293T cells. At 48 h post-transfection, 5% normal goat serum was used to block the cells, and then the blocked cells were incubated with FITC-conjugated recombinant soluble Cd2 protein (FITC-Cd2) at different concentrations (1, 5, and 10 µg/mL) (31). FCM analysis was performed as described above.

### Statistical Analysis

All data are presented as the mean ± SD. Statistical evaluation of differences between means of experimental groups was performed using ANOVA and multiple Student’s *t*-tests. Survival curve differences in the immunoprotection assay were assessed using the log-rank test. Both *p* values <0.05 and <0.01 were considered to be significant. The sample number for each group of fish exceeded 30. All experiments were replicated at least three times.

## Results

### Identification of *cd58* and *cd2* Genes

**Figure 1:**
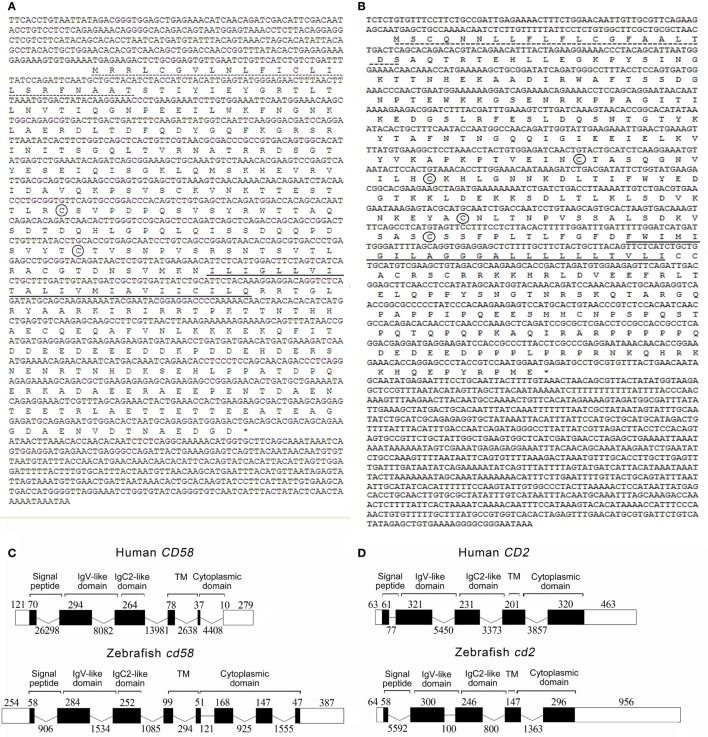
**(A,B)** The cDNA sequences and deduced amino acids of *cd58 and cd2*. The signal peptide and transmembrane domain are underlined with the dotted and solid lines, respectively. Two conserved cysteines in Cd58 and four in Cd2 amino acid sequences forming IgC2-like domain structures are encircled, respectively. The asterisk represents the stop codon. **(C,D)** Comparison of the intron/exon organizations of the *CD58* and *CD2* genes in humans and zebrafish. Exons and introns are shown with the black boxes and lines, and their sizes are indicated by the numbers found above and below the sequences, respectively. Black and white areas indicate the coding regions and untranslated regions, respectively. Schematic diagrams were included above the exon/intron organization cartoons to indicate the exons in terms of protein domains, including signal peptide, IgV domain, IgC2-like domain, transmembrane domain (TM), and cytoplasmic domain.

Through systematic searches of the zebrafish amino acid sequences in the zebrafish protein databases using the human and porcine CD58 and CD2 sequences, the corresponding homologous of zebrafish *cd58* and *cd2* were retrieved. The cloned *cd58* cDNA consists of 1,751 bp with a 254-bp 5′ untranslated region (UTR), a 1,110-bp ORF encoding 369 amino acids, and a 387-bp 3′UTR (GenBank accession No. MG571530). The *cd2* cDNA is 2,070 bp in length containing a 64-bp 5′UTR, a 1,050-bp ORF encoding 349 amino acids, and a 956-bp 3′UTR (GenBank accession No. MG571531) (Figures 1A,B). By comparing *cd58* and *cd2* cDNA sequences with the matching genomic sequences, the organization of *cd58* and *cd2* genes was clearly elucidated (Figures 1C,D). Results showed that the *cd58* gene comprised 8 exons and 7 introns and was located within an 8.4-kb genomic fragment on zebrafish chromosome 9. Genes adjacent to the human CD58 gene on chromosome 1 (51), such as *nhlh2, igsf3, ttf2, ptgfrn, gdap2*, and *wdr3*, were all found to be clustered with the *cd58* gene on chromosome 9, and they shared an overall conserved chromosome synteny between humans and zebrafish, although the synteny of *nhlh2 and igsf3* loci was in a reverse order (Figure S1A in Supplementary Material). The exon organization and encoding sequences for functional domains in the *cd58* gene resemble those of the CD58 gene, among which exons 1–2, 2–3, 3, and 4–5 of both *cd58* and CD58 genes are predicted to encode the signal peptides, Ig variable region-like (IgV-like), Ig constant region-like (IgC2-like), and the transmembrane domains, respectively (Figure 1C). Notably, the *cd58* gene only differs from the CD58 gene in terms of the latter’s lack of exons 7 and 8, which were predicted to encode an extra intracellular region (126 amino acids) at the carboxyl terminal of the Cd58 protein. It allows Cd58 to have a longer intracellular domain in the cytoplasm. Unlike the human CD2 gene located on chromosome 1 downstream adjacent to the CD58 gene (52), the *cd2* gene is separately distributed within a 9.3-kb genomic fragment on zebrafish chromosome 1. However, the structural organization of the *cd2* gene is highly conserved with the CD2 counterpart. Both *cd2* and CD2 genes contain 5 exons and 4 introns, among which exons 1–2, 2–3, 3–4, 4, and 4–5 of both *cd2* and CD2 genes are predicted to encode the signal peptides, IgV-like domain, IgC2-like domain, transmembrane domain, and cytoplasmic region, respectively (Figure 1D). Because genomic information needed for annotation of the immediate neighbor genes around *cd2* locus is limited in current zebrafish genome database, detailed synteny organization for *cd2* gene will not be clarified until the genomic database is further improved.

### Characterization of Cd58 and Cd2 Structures

Both Cd58 and Cd2 are predicted to be the type I transmembrane proteins with typical structural features of the IgSF and a molecular mass of 41.48 and 39.02 kDa, respectively. They have four major functional domains, including an extracellular IgV-like domain (23–116 amino acids for Cd58; 33–121 amino acids for Cd2), an IgC2-like domain (130–187 amino acids for Cd58; 123–203 amino acids for Cd2), a transmembrane domain (208–230 amino acids for Cd58; 215–237 amino acids for Cd2), and an intracellular tail. Multiple sequence alignments show that the Ig domains and functional amino acid residues of Cd58 and Cd2 share high degree of homology with each molecule in higher vertebrates (Figures S2A,B in Supplementary Material). For example, the IgV-like domain of Cd58, whose homology is sufficient for CD58 binding to CD2 in mammals, shares high amino acid identity with that of humans (52%) and other mammalian counterparts (41–52%). Furthermore, most of the key amino acid residues of CD58 involved in electrostatic interactions or hydrogen bonds at the interface of the CD58–CD2 complex in humans and other mammalian species are also conserved in the Cd58 protein, which include Glu-46, Trp-49, Lys-50, Lys-55, Glu-58, and Glu-99. The IgV-like domain of Cd58 and Cd2 does not contain a disulfide bond, whereas the IgC2-like domain of Cd58 has one disulfide bond and Cd2 has two disulfide bonds, which are also similar from fish to mammals. Moreover, mammalian CD58 and CD2 were found to be highly glycosylated (53, 54). Consistently, four N-linked glycosylated sites (Asn-37, Asn-87, Asn-129, and Asn-190) can be predicted in Cd58, including two inside the IgV-like domain, one inside the IgC2-like domain, and one outside the IgC-like domain. In parallel, the extracellular domain of Cd2 contains three potential glycosylated sites (Asn-60, Asn-131, and Asn-186): one inside the IgV-like domain and two inside the IgC2-like domain. These distributions of potential N-linked glycosylated sites in Cd58 and Cd2 are conserved to their mammalian counterpart. Moreover, the intracellular PPLPRPR motif of mammalian CD2, which contributes to the recruitment of downstream adaptor proteins (such as CD2AP and CD2BP1) once CD2 binds to CD58, was also found in the Cd2 homolog (Figure S2B in Supplementary Material). Phylogenetic analysis showed that Cd58 and Cd2 were closely clustered to their homologs in different species with high bootstrap probability, in which CD48, CD150, and Ly-9 with the closest genetic relationship to CD58 and CD2 were included as well (Figure S1B in Supplementary Material).

**Figure 2:**
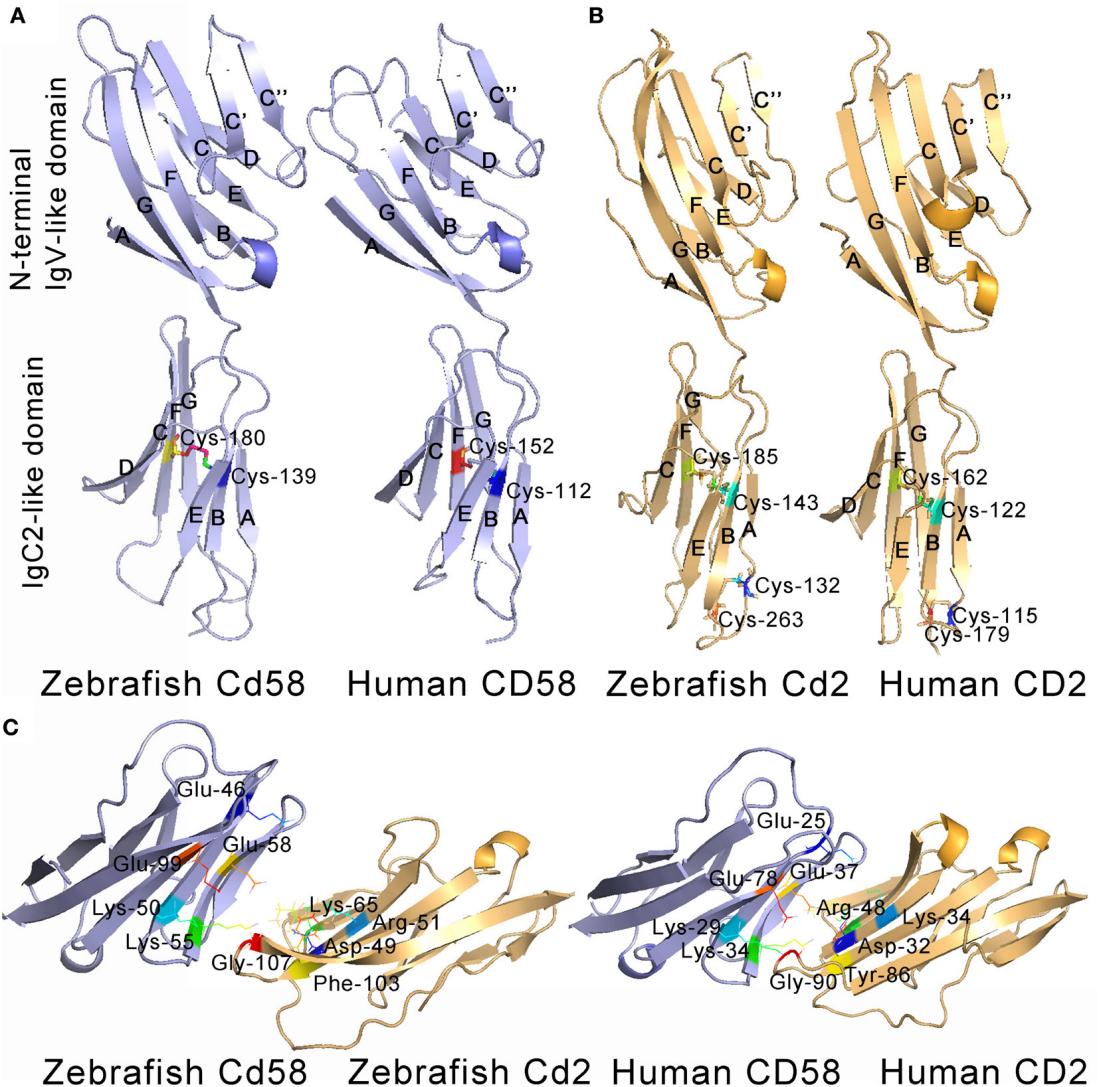
Tertiary structures of the extracellular domains of CD58 **(A)** and CD2 **(B)**. The structures were predicted by homology modeling with human CD58 (PDB 1CCZ) and CD2 (PDB 1HNF) extracellular domains as templates, showing the overall homology with human counterparts. The β strands of N-terminal IgV-like domains (AGFCC′C″BED) and the membrane-proximal IgC2-like domains (AEBDCFG and AEBCFG) of CD58 (blue) and CD2 (yellow) are labeled. **(C)** Detailed views of anti-parallel β-strands in stacked pleated β-sheets, α-helix structures, hydrophobic loops, Cys residues, and residues across the CD2–CD58 interface are depicted and labeled.

Given the pivotal roles of the extracellular IgV-like and IgC2-like domains in CD58 and CD2 interaction and functional activities, these two domains were selected for further tertiary structure analysis. Through homologous modeling using human CD58 and CD2 extracellular domains as templates, the N-terminal IgV-like domains of Cd58 and Cd2 were found to be folded with two antiparallel β sheets formed by nine β strands (AGFCC′C″BED), whereas the membrane-proximal IgC2-like domains of Cd58 and Cd2 were equally folded with two antiparallel β sheets formed by seven and six β strands (AEBDCFG and AEBCFG), respectively. In addition, both Cd58 and Cd2 contain one α-helix (between E and F strands) and several hydrophobic loops in their IgV-like domains. These structural features are highly identical to their human counterparts (Figure 2). However, there are still some imperceptible differences between zebrafish CD2/CD58 and human CD2/CD58. For example, the α-helix between C and C′ strands contained in the CD2 IgV-like domain is lost in Cd2. Except for the canonical inter-sheet disulfide bond (Cys-143–Cys-185) between B and F strands in CD2 IgV-like domain, there is another disulfide bond (Cys-132–Cys-263) between the ends of the A and G strands in Cd2. The IgC2 domain of Cd58 contains two inter-Cys residues (Cys-139 and Cys-180), which can potentially form one disulfide bridge between the B and F strands. On the other hand, the key residues mediating the adhesion function of mammalian CD58 and CD2 are located on the GFCC′C″ face of the IgV-like domain, such as Glu-25, Lys-29, Lys-34, Glu-37, and Glu-78 of CD58 and Lys-43, Asp-32, Arg-48, Lys-34, Tyr-86, and Gly-90 of CD2 (5). Most of these key residues are conserved in zebrafish, such as Glu-46, Trp-49, Lys-50, Lys-55, Glu-58, and Glu-99 of Cd58 and Lys-43, Asp-49, Arg-51, Lys-65, Phe-103, and Gly-107 of Cd2 (Figure 2C). Interestingly, Tyr-86, an important residue in mammalian CD58 and CD2 interaction, is replaced by Phe-103 in Cd2, which can also be observed in other fish species, such as *Oreochromis niloticus* (4, 5, 55). Coincidentally, a research in humans has proved that the mutation from Tyr to Phe at site 86 of CD2 has no effect on the interaction between CD58 and CD2. Thus, these two residues (Tyr and Phe) functioning in CD58 and CD2 interaction can be substituted for each other in different species during evolution. Overall, the extracellular domains of Cd58 and Cd2 are well conserved to their human counterparts.

### Preparation of siRNA-Encoding LV and Abs

**Figure 3:**
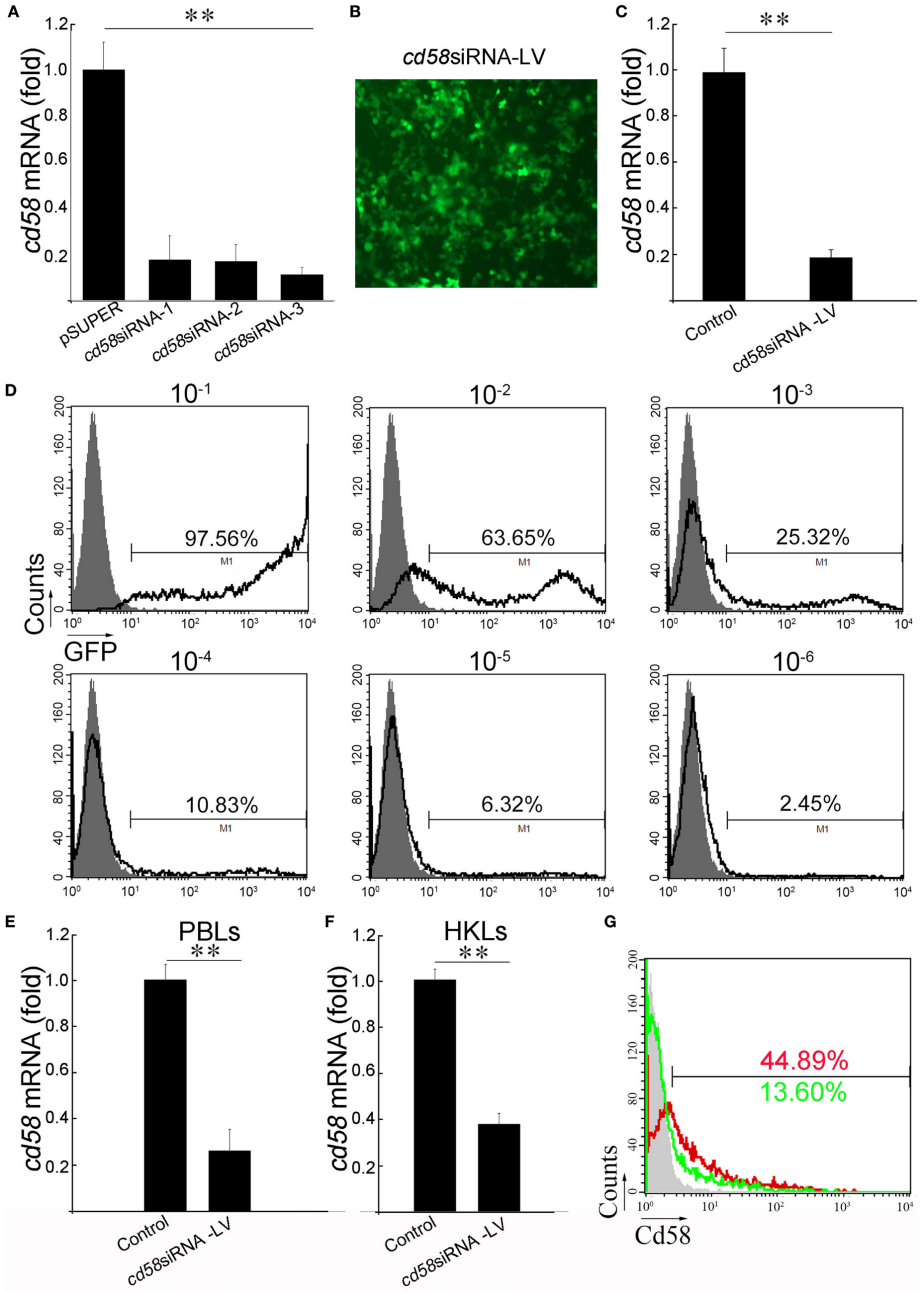
*In vivo* knockdown assay of *cd58*. **(A)** Screening of the most effective small interfering RNA (siRNA) interfering zebrafish *cd58* expression. Three designed siRNAs encoding DNA oligonucleotides targeting different regions of each zebrafish *cd58* mRNA were constructed to pSUPER vectors (*cd58*siRNA-1–3), which were in turn co-transfected with overexpression pcDNA6-*cd58* plasmid into HEK293T cells, respectively. Inhibitory efficiencies were measured by real-time PCR. **(B)** After choosing the most effective siRNA targeting CD58, an LV harboring the siRNA was produced. The infection ability of the constructed LV was evaluated by detecting the GFP fluorescence release in HEK293T cells under a fluorescent microscope (Zeiss Axiovert 40 CFL; Zeiss, Jena, Germany) with original magnification at 400×. Scale bars, 200 µm. **(C)** The interfering abilities of the LV were examined in HEK293T cells transfected with the target gene overexpression plasmid *in vitro* by real-time PCR analyses. **(D)** The LV titer was assessed by the percentage of GFP^+^ HEK293T cells after exposure to different dilutions of LVs using flow cytometry (FCM). The gray histograms show background fluorescence on control cells without LV infection. The numbers above the bracketed lines indicate the percentage of cells in each. Data are from three independent experiments. **(E–G)** Detection of the inhibitory effect of CD58siRNA-LV by *in vivo* administration through real-time PCR and FCM analyses. The expression of *cd58* in peripheral blood leukocytes (PBLs) and head kidney lymphocytes (HKLs) was detected using real-time PCR **(E,F)**. For FCM analysis, the scrambled siRNA-LV (red) or *cd58*siRNA-LV (green) treated leukocytes was labeled with FITC-anti-Cd58. Scrambled siRNA-LV was set as control. Mean ± SE of results from three independent experiments are shown (*p < 0.05, **p < 0.01).

To prepare the siRNA-encoding LV against *cd58* for functional investigation, three siRNAs (*cd58*siRNA-1–3) targeting different regions of *cd58* were predicted by a template design program. Of the three candidates, *cd58*siRNA-3 is the highest effective siRNA to induce *cd58* mRNA degradation (Figure 3A). Thus, *cd58*siRNA-3 was used to construct the *cd58* siRNA-encoding LV (*cd58*siRNA-LV). The infectiousness and interference of *cd58*siRNA-LV were assessed in HEK293T cells by using a GFP-based detecting system, FCM analysis, and real-time PCR *in vitro* (Figures 3B–D). Results showed that the titer of the LV reached 10^5^ TU/μL with an interference efficacy above 81%. For *in vivo* knockdown evaluation, healthy fish were i.p. with *cd58*siRNA-LV or Scrambled siRNA-LV thrice with a 24-h time interval, respectively. The real-time PCR results showed that the expression levels of *cd58* were strongly inhibited in PBLs and HKLs (Figures 3E–G). As shown in Figure 3G, compared to the scrambled siRNA-LV (red) treated leukocytes, the expression levels of Cd58 in the *cd58*siRNA-LV (green) treated leukocytes was strongly inhibited [decreased from 44.89 ± 3.32 to 13.60 ± 1.96% (*p* < 0.05)].

Antibodies against Cd58 (anti-Cd58) and Cd2 (anti-Cd2) were prepared based on the epitope (YGRTLTLNVTIQGNPE) prediction and recombinant protein production of these two molecules (Figure S3A in Supplementary Material). By using affinity purification, the anti-Cd58 and anti-Cd2 Abs were isolated from the immunized mouse/rabbit sera into IgG isotypes and detected to have high specificities to Cd58 and Cd2 proteins with average titers above 1:10,000 by ELISA and Western blot analyses (Figures S3B,C in Supplementary Material).

### Subcellular Localization of Cd58 and Cd2

**Figure 4:**
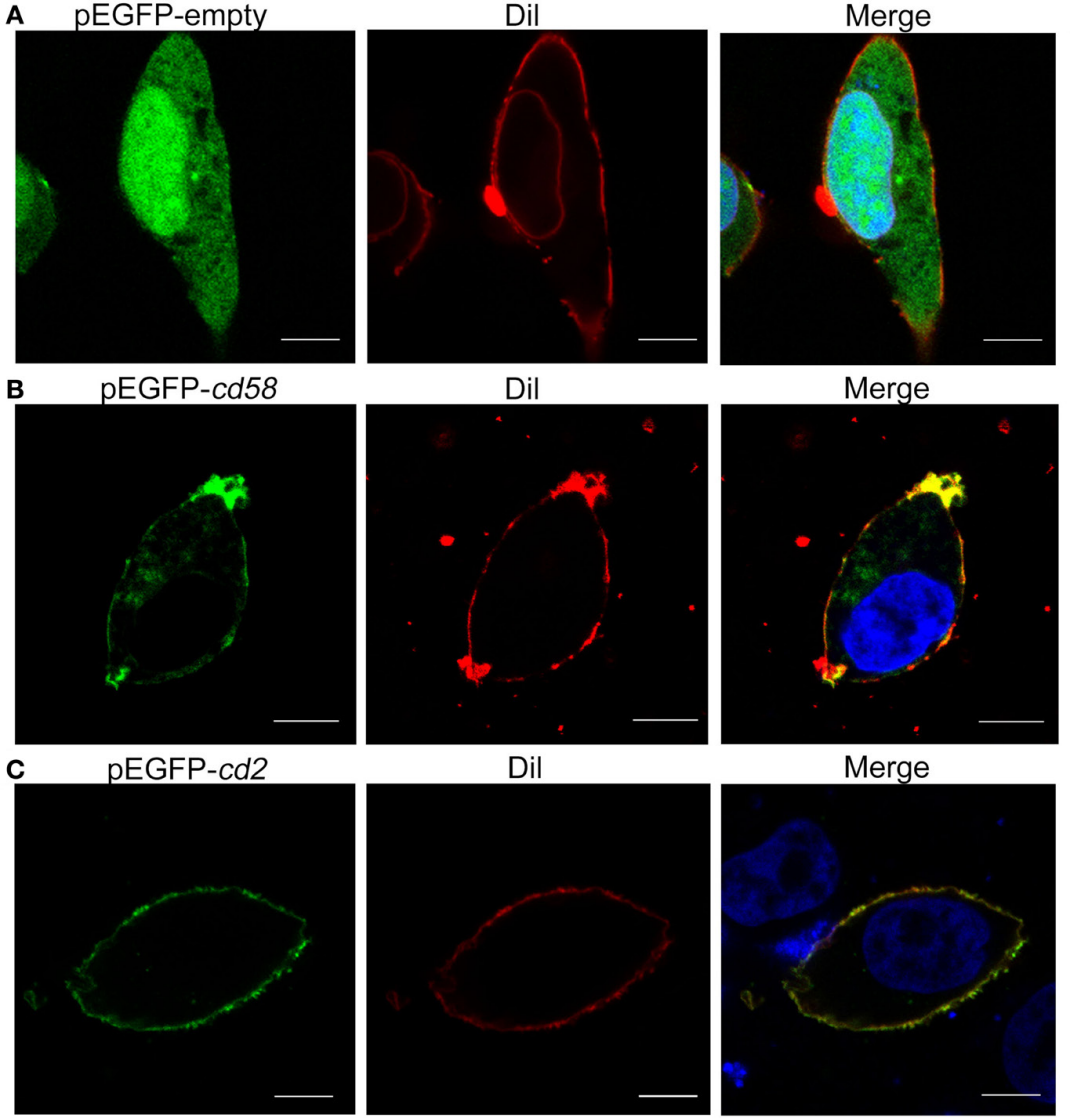
Subcellular localization analyses of Cd58 **(B)** and Cd2 **(C)** in HEK293T cells. Cells were transfected with empty control **(A)** or pEGFP-*cd58* and pEGFP-*cd2* **(B,C)** plasmids. Cd58 and Cd2 were clearly located on the cell surface membranes. Images were captured under a two-photon laser scanning confocal microscope (Zeiss LSM-710; original magnification, 630×). Scale bars, 10 µm.

For subcellular localization analysis, the pEGFPN1-*cd58*, pEGFPN1-*cd2*, and control pEGFPN1 constructs were transfected into HEK293T cells. The overexpressed Cd58 and Cd2 fusion proteins all clearly displayed dot-like signatures on the cell surface, which merged well with the DiI-stained membrane, whereas the control GFP protein was randomly distributed both in the cytoplasm and nucleus. The results suggest that Cd58 and Cd2 are membrane proteins (Figure 4). Similar membrane localization of Cd58 and Cd2 was also seen in Mhc-ii^+^ APC and Cd4^+^ T cells.

### Tissue and Cellular Distribution of Cd58 and Cd2

**Figure 5:**
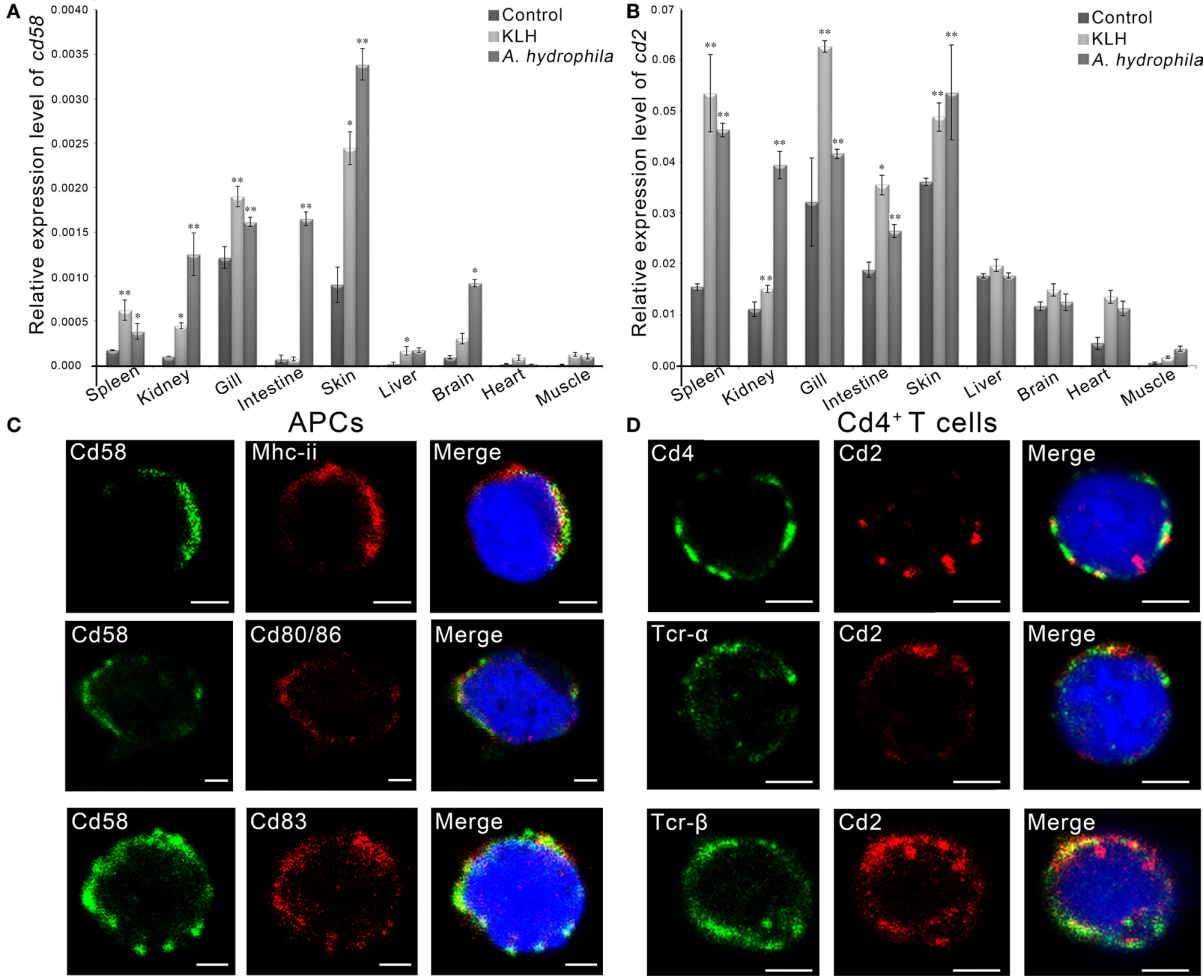
Tissue and cellular distribution analyses of Cd58 and Cd2. **(A,B)** Tissue distribution analyses of *cd58* **(A)** and *cd2* **(B)** in control fish or in antigen [keyhole limpet hemocyanin (KLH) or *Aeromonas hydrophila*] challenged fish by real-time PCR. The relative gene expression profiles of *cd58* and *cd2* in various tissues were displayed as relative values to the expression levels of β-actin (*cd58*/β-actin and *cd2*/β-actin), respectively. Each sample was obtained from 10 fish and run in triplicate parallel reactions. The experiments were repeated independently at least three times (*p < 0.05, **p < 0.01). **(C,D)** Immunofluorescence staining of the leukocytes separated from the blood, spleen, and kidney tissues of the fish stimulated with KLH (in combination with lipopolysaccharide). Cells were stained with rabbit anti-Cd58 together with mouse anti-MHC class II (Mhc-ii), mouse anti-Cd80/86, or mouse anti-Cd83 **(C)**, or stained with mouse anti-Cd2 together with rabbit anti-Cd4, rabbit anti-Tcr-α, or rabbit anti-Tcr-β **(D)**. Nonrelated mouse and rabbit IgG isotypes were used as negative controls (data not shown). DAPI stain shows the location of the nuclei. The images were obtained using a two-photon laser scanning confocal microscope (Zeiss LSM-710; original magnification, 630×). An enlarged image of the target cell was generated by fourfold magnification. Scale bars, 2 μm.

By real-time PCR, it was found that the *cd58* and *cd2* mRNAs were expressed in almost all tissues examined, including the skin, gill, liver, kidney, spleen, intestine, heart, brain, and muscle (Figures 5A,B). Upon *in vivo* antigen (KLH or *A. hydrophila*) stimulation, the expression level of both *cd58* and *cd2* could be significantly upregulated (*p* < 0.05), particularly in immune-relevant tissues, such as the spleen, skin, and gill (Figures 5A,B). For functional evaluation of *cd58* and *cd2* in adaptive humoral immunity, double-immunofluorescence staining was performed to detect their distributions on APC and Cd4^+^ T cells. Results indicated that Cd58 could clearly be co-localized with Mhc-ii, Cd80/86, and Cd83, three hallmark molecules of the APC (Figure 5C); while Cd2 could be co-localized with Cd4, Tcr-α, and Tcr-β molecules (Figure 5D). These observations revealed that Cd58 and Cd2 were expressed on Mhc-ii^+^Cd80/86^+^Cd83^+^ APCs and Cd4^+^ αβ T cells, respectively, whose interaction may play important roles in antigen-induced adaptive immune responses.

### Upregulation of *cd58* and *cd2* by Ag Stimulation

**Figure 6:**
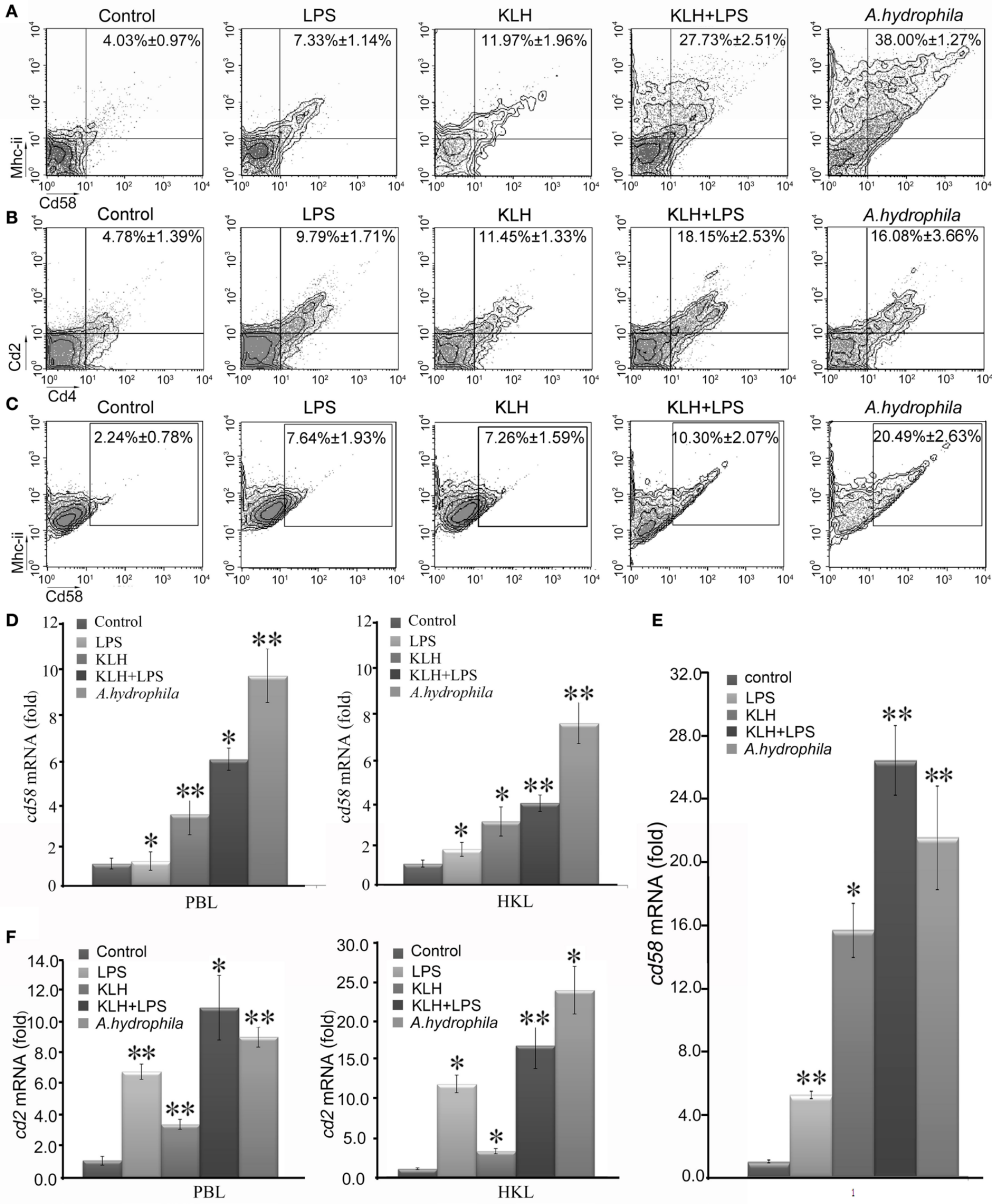
Induced expression analysis of *cd58* and *cd2* on APCs or Cd4^+^ T cells upon keyhole limpet hemocyanin (KLH) [plus lipopolysaccharide (LPS)] or *Aeromonas hydrophila* stimulations. **(A,B)** Flow cytometric analysis of Cd58 or Cd2 expression level on APCs or Cd4^+^ T cells, which were sorted from peripheral blood, spleen, and kidney tissues 3 days after i.p. stimulation with PBS, KLH, LPS, KLH plus LPS, or *A*. *hydrophila*. The numbers above the outlined areas indicate the percentage of double positive cells in each group. **(C)** Flow cytometric analysis of Cd58^+^Mhc-ii^+^ cells from sorted APCs upon pulsing with PBS, KLH, LPS, KLH plus LPS, or *A*. *hydrophila* for 8 h *in vitro*. The numbers above the outlined areas indicate the percentage of double positive cells in each group. Means ± SE of results from three independent experiments are shown (*p < 0.05, **p < 0.01). **(D)** Real-time PCR analysis for the expression of Cd58 in APCs of each *in vivo* treatment group. **(E)** Real-time PCR analysis for the expression of *cd58* in APCs with *in vitro* treatment of PBS, KLH, LPS, KLH plus LPS, or *A*. *hydrophila*. **(F)** Real-time PCR analysis for the expression of *cd2* in Cd4^+^ T cells of each *in vivo* treatment group. The relative expression values were averaged from the data in three parallel reactions, and the results were obtained from at least three independent experiments (*p < 0.05, **p < 0.01).

To provide initial insight into the role of Cd58 and Cd2 in antigen-induced adaptive immunity, the upregulation of *cd58* and *cd2* in response to Ag stimulation was examined *in vivo* by FCM and real-time PCR. Results showed that the expression levels of Cd58 on Mhc-ii^+^ cells and Cd2 on Cd4^+^ T cells were induced by different antigen stimulations, as determined by the significant increase (*p* < 0.01) of the percentages of Mhc-ii^+^Cd58^+^ or Cd4^+^Cd2^+^ cells in PBLs and HKLs stimulated by KLH, LPS, KLH plus LPS, or *A. hydrophila*, compared with those of mock PBS-treated fish (Figures 6A,B). Among the four stimulated groups, the *A. hydrophila*-stimulated and KLH plus LPS groups had the most striking upregulation of Mhc-ii^+^Cd58^+^ cells (increased by 38.00 ± 1.27%) and Cd4^+^Cd2^+^ cells (increased by 18.15 ± 2.53%). Meanwhile, the KLH plus LPS costimulated group had a more striking upregulation of Mhc-ii^+^Cd58^+^ cells (increased by 27.73 ± 2.51%) than the sum of the KLH- or LPS-treated alone groups (increased by 11.97 ± 1.96 or 7.33 ± 1.14%, respectively). The enhanced expression of *cd58* and *cd2* upon antigen stimulation was also detected by real-time PCR (Figures 6D,F). Similarly, by *in vitro* stimulating the sorted Mhc-ii^+^ cells (from PBLs and HKLs) with KLH, LPS, KLH plus LPS, or *A. hydrophila*, the expression of *cd58* was determined to be significantly upregulated (*p* < 0.05) in the Mhc-ii^+^ cells by real-time PCR, and accordingly, the percentage of the Mhc-ii^+^Cd58^+^ cells was dramatically increased (*p* < 0.01) by FCM analysis (Figures 6C,E). These findings suggest that *cd58* and *cd2* can be upregulated in/on Mhc-ii^+^ or Cd4^+^ T cells by antigen stimulation. This behavior is in agreement with the characteristics of Cd58 and Cd2 as functional molecules on APC or Cd4^+^ T cells.

### *In Vitro* Evaluation of Cd58 and Cd2 in Cd4^+^ T Cell Activation

**Figure 7:**
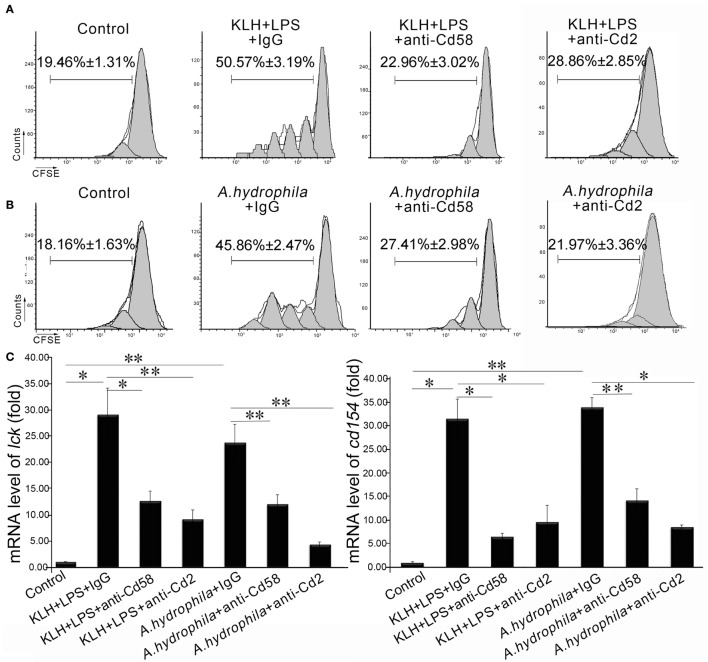

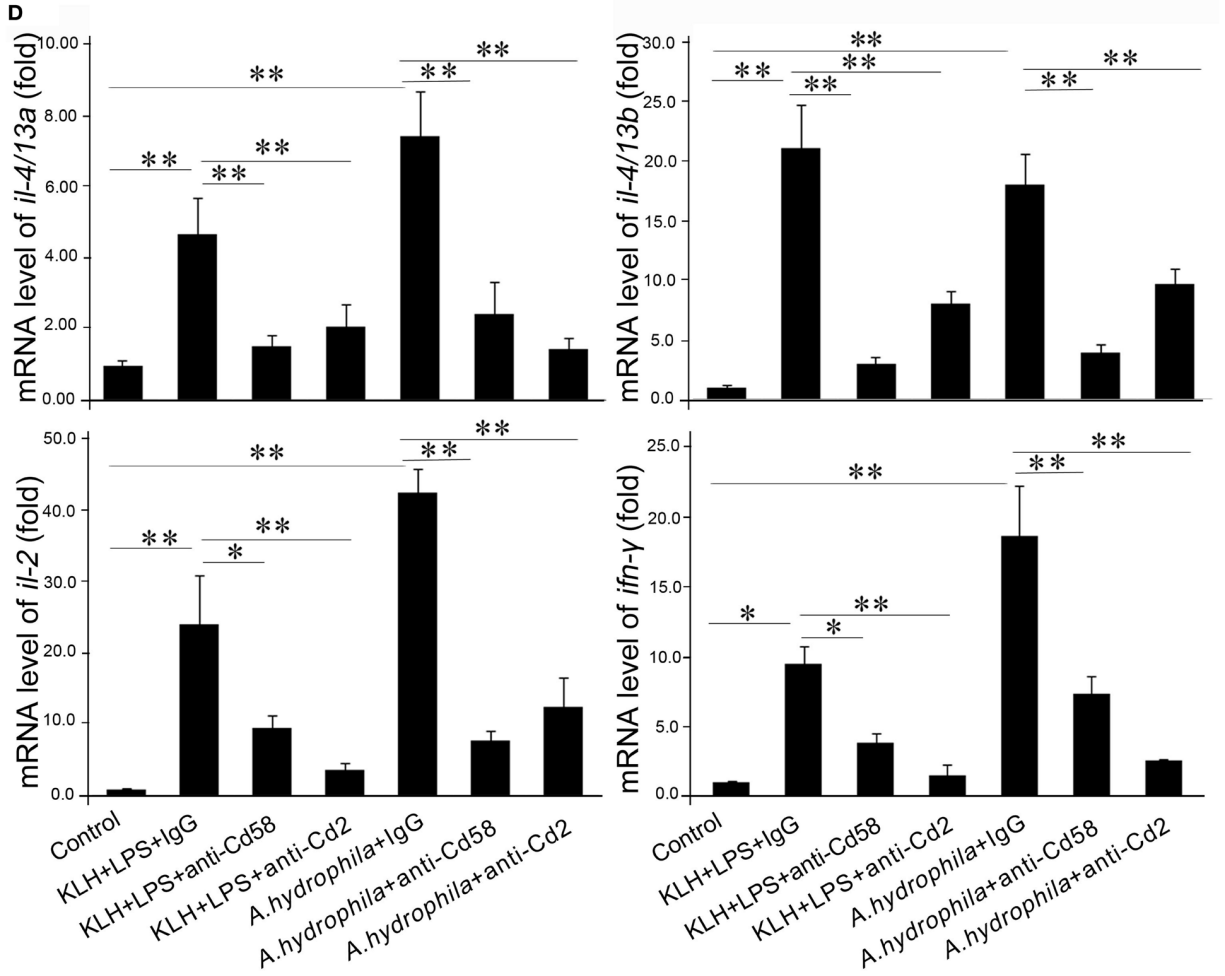
*In vitro* evaluation of Cd58 and Cd2 in APC-initiated Cd4^+^ T cell proliferation. The proliferation of Cd4^+^ T_KLH_ **(A)** or Cd4^+^ T_A.h_ **(B)** was inhibited by treating the cells with anti-Cd58 or anti-Cd2 antibodies, determined by CFSE dilution through flow cytometry and by the expression levels of *lck* and *cd154* **(C)** and cytokines (*il-4/13a, il-4/13b, il-2*, and *ifn-γ*) production **(D)** through real-time PCR. Cd4^+^ T_KLH_ cells or Cd4^+^ T_A.h_ co-cultured with PBS-loaded primary APCs were used as control. Error bars represent SE. All data were from at least three independent experiments (*p < 0.05, **p < 0.01).

To evaluate the function of Cd58 and Cd2 in APC-initiated Ag-specific T-cell activation, an *in vitro* blockade assay was performed by using anti-Cd58 and anti-Cd2 Abs. The sorted Mhc-ii^+^ APCs were stimulated with soluble (KLH plus LPS) or particulate (*A. hydrophila*) antigens; treated with anti-Cd58 Ab, anti-Cd2 Ab, or nonrelated rabbit IgG; followed by incubation with antigen-specific Cd4^+^ T cells (Cd4^+^ T_KLH_ or Cd4^+^ T*_A_*_._*_h_*). Proliferation and activation of the responder Cd4^+^ T cells were assessed by CFSE dilution and real-time PCR. As shown in Figures 7A,B, the proliferation of Cd4^+^ T_KLH_ or Cd4^+^ T*_A_*_._*_h_* in response to KLH- or *A. hydrophila*-loaded APCs significantly decreased (*p* < 0.01) in the blockade groups compared with that in the control groups. Correspondingly, the activation of Cd4^+^ T cells was determined by the expression of *lck* and *cd154*. The expression levels of *lck* and *cd154* in the blockade co-cultures were significantly downregulated (*p* < 0.05) (Figure 7C). In addition, the blockade of *cd58* and *cd2* significantly inhibited (*p* < 0.05 or *p* < 0.01) the expression of the Th2-typic hallmarks (*il-4/13a* and *il-4/13b*) and the Th1-typic ones (*ifn-γ* and *il-2*) in Cd4^+^ cells (Figure 7D). These results suggest that *cd58* and *cd2* are essential for the activation of Cd4^+^ T cells in adaptive immunity.

### *In Vivo* Evaluation of Cd58 in Cd4^+^ T Cell Activation

**Figure 8:**
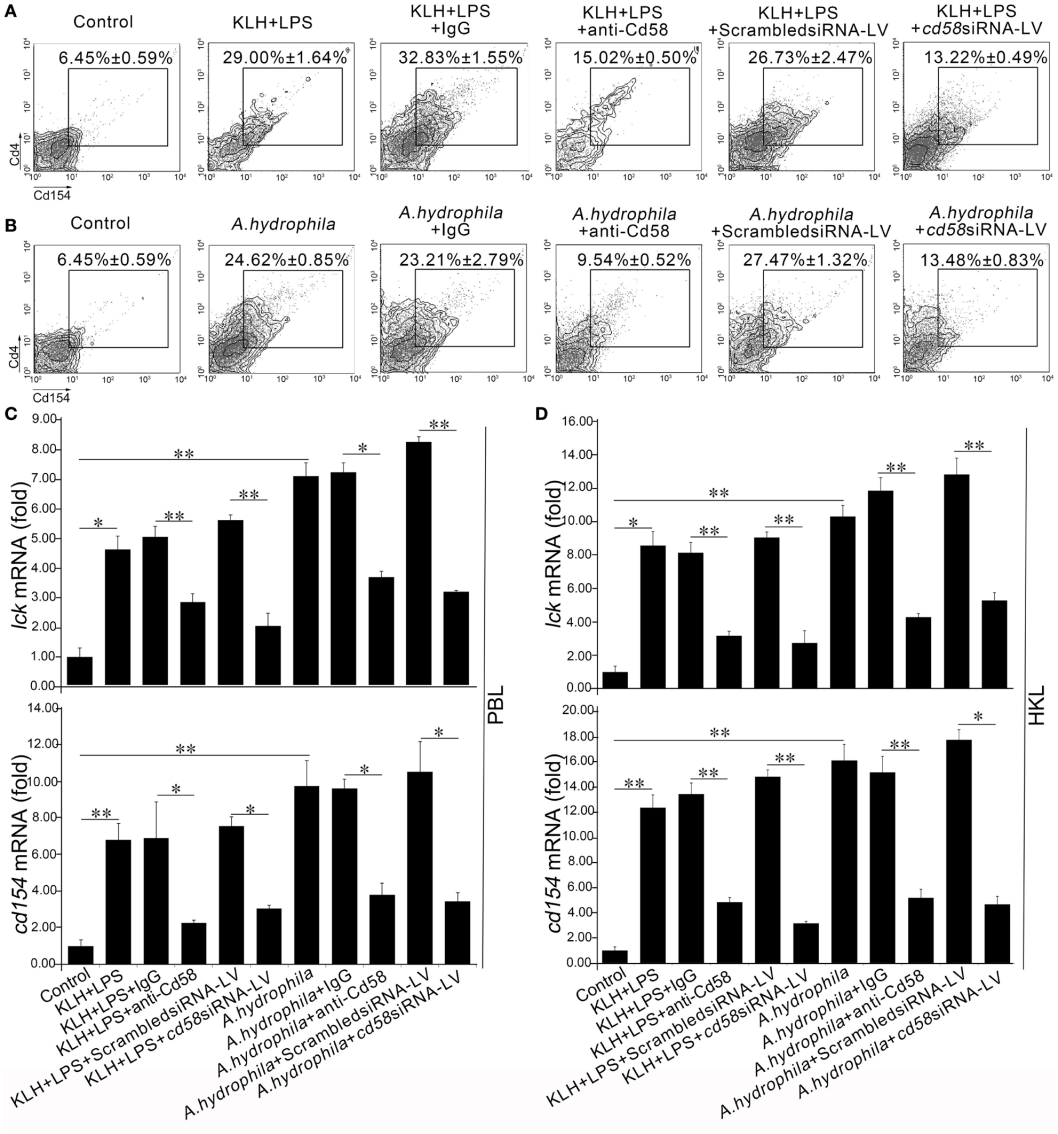
*In vivo* evaluation of Cd58 in Cd4^+^ T cell activation. The degree of Cd4^+^ T cell activation is represented by the percentage of Cd4^+^Cd154^+^ T cells determined by flow cytometry **(A,B)** and by the expression levels of *lck* and *cd154* genes detected by real-time PCR **(C,D)**. In the flow cytometric analysis, different treatments were presented at the top of each block diagram. The numbers adjacent to the outlined areas indicate the percentage of Cd4^+^Cd154^+^ cells in each treatment group. In the real-time PCR assay, PCRs were run in combination with the endogenous β-actin control. Error bars represent SE. All data are from at least three independent experiments (*p < 0.05, **p < 0.01).

To further determine the costimulatory role of Cd58 in APC-initiated Ag-specific Cd4^+^ T cell activation, *in vivo* Ab-mediated blockade and LV-mediated knockdown assays were conducted by i.p. administration of anti-Cd58 Ab and *cd58*siRNA-LV. Results showed that the activation of antigen-specific Cd4^+^ T cells was significantly inhibited (*p* < 0.01) in the Cd58 blockade (anti-Cd58 Ab administered) groups compared with that of the non-blockade control (normal immunized) group as the proportion of the activated Cd4^+^ T cells upon stimulation of KLH plus LPS (Cd4^+^Cd154^+^ T_KLH_) or *A. hydrophila* (Cd4^+^Cd154^+^ T*_A_*_._*_h_*) declined from 29.00 ± 1.64 to 15.02 ± 0.50% and from 24.62 ± 0.85 to 9.54 ± 0.52%, respectively (Figures 8A,B). By contrast, no significant reductions in the percentages of Cd4^+^Cd154^+^ T_KLH_ (32.83 ± 1.55%) and Cd4^+^Cd154^+^ T*_A.h_* (23.21 ± 2.79%) were observed in the nonrelated IgG-treated (negative control) groups compared with those of the non-blockade control groups. Similarly, the percentages of Cd4^+^Cd154^+^ T_KLH_ and Cd4^+^Cd154^+^ T*_A_*_._*_h_* in the LV-mediated knockdown groups were dramatically decreased (*p* < 0.05) from 26.73 ± 2.47% (KLH and LPS plus scrambled siRNA-LV group) to 13.22 ± 0.49% (KLH and LPS plus *cd58*siRNA-LV group) and from 27.47 ± 1.32% (*A. hydrophila* plus scrambled siRNA-LV group) to 13.48 ± 0.83% (*A. hydrophila* plus *cd58*siRNA-LV group) (Figures 8A,B), respectively. Furthermore, the expression levels of *lck* and *cd154* (upon KLH plus LPS or *A. hydrophila* stimulation) in PBLs and HKLs were remarkably (*p* < 0.05) downregulated in the Ab-mediated blockade and LV-mediated knockdown groups (Figures 8C,D). These results provide *in vivo* evidence that Cd58 is essential to activate Cd4^+^ T cells in response to different antigens.

### Effects of Cd58 and Cd2 on B Cell Activation and Ab Production

**Figure 9:**
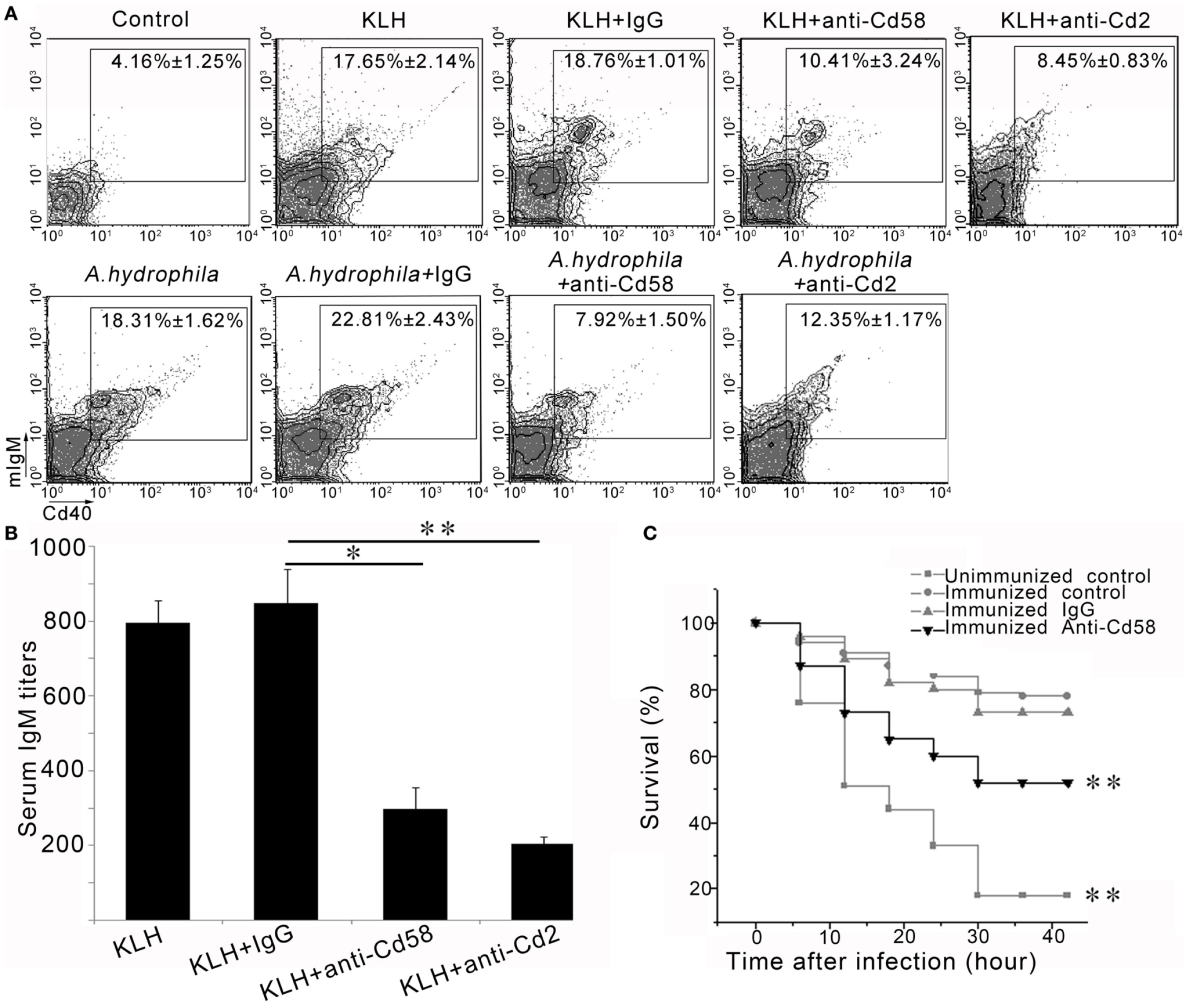
*In vivo* evaluation of Cd58 and Cd2 in B cell activation, antibody production and vaccinated immunoprotection. **(A)** Involvement of Cd58 and Cd2 in B cell activation. The degree of B cell activation is represented by the percentage of mIgM^+^Cd40^+^ cells determined by flow cytometry. The experimental treatments are presented at the top of each block diagram. The data above the outlined area in each block diagram indicate the average percentage of mIgM^+^Cd40^+^ B cells in each treatment group. **(B)** Involvement of Cd58 and Cd2 in IgM production. The titer of IgM against keyhole limpet hemocyanin (KLH) in each treatment group was examined by ELISA (*n* = 150) (*p < 0.05, **p < 0.01). **(C)** Blockade of Cd58 impairs the vaccinated immunoprotection against bacterial (*Aeromonas hydrophila*) challenge. Data points are from three independent experiments (*n* = 30); Differences were analyzed using log-rank test (**p < 0.01).

To further address the role of Cd58 and Cd2 in adaptive humoral immunity, the involvement of Cd58 and Cd2 in Cd4^+^ T-cell-initiated B-cell activation and Ab production was examined. *In vivo* inhibition assays for B-cell activation and Ab (IgM) production were conducted after blockade of Cd58 or Cd2 by anti-Cd58 Ab or anti-Cd2 Ab, respectively. As shown in Figure 9A, upon the administration of KLH or *A*. *hydrophila*, the percentage of the activated mIgM^+^Cd40^+^ B cells (mIgM^+^Cd40^+^ B_KLH_; mIgM^+^Cd40^+^ B*_A.h_*) in the Cd58 blockade groups significantly declined (*p* < 0.01) from 17.65 ± 2.14 to 10.41 ± 3.24% (for mIgM^+^CD40^+^ B_KLH_) and from 18.31 ± 1.62 to 7.92 ± 1.50% (for mIgM^+^Cd40^+^ B*_A.h_*) compared with that of the non-blockade control (normal immunized) group. Correspondingly, Cd2 blockade groups significantly declined (*p* < 0.01) from 17.65 ± 2.14 to 8.45 ± 0.83% (for mIgM^+^Cd40^+^ B_KLH_) and from 18.31 ± 1.62 to 12.35 ± 1.17% (for mIgM^+^Cd40^+^ B*_A.h_*). By contrast, no significant decline in the percentages of mIgM^+^Cd40^+^ B_KLH_ (18.76 ± 1.01%) and mIgM^+^Cd40^+^ B_A.h_ (22.81 ± 2.43%) was observed in the nonrelated IgG-treated groups (Figure 9A). Accordingly, the production of serum IgM against KLH in the Cd58 or Cd2 blockade group was significantly reduced (*p* < 0.05) compared with that in the nonrelated IgG-administered control group (Figure 9B). These results support the costimulatory function of Cd58 and Cd2 in adaptive humoral immunity, which contributes to the full activation of Cd4^+^ T cells and subsequent B cells, as well as the production of the Ab.

### Functional Evaluation of Cd58 by a Vaccinated Immunoprotection Assay

To verify that Cd58 acts as a costimulatory molecule involved in the initiation of adaptive humoral immunity, a functional evaluation was performed by the impairment of a vaccinated immunoprotection *via* blockade of Cd58 during the vaccination by using an inactivated *A*. *hydrophila* vaccine. Results showed that 18.7 ± 1.65 and 83.4 ± 2.98% (*p* < 0.01, log-rank test) of the fish in the unimmunized negative control group (without vaccination) and the immunized positive control group (received vaccination) survived after the virulent *A*. *hydrophila* challenge, respectively (Figure 9C). This result indicated that the adaptive immunity was well established after vaccination. However, the survival rates in the Cd58 blockade group (received vaccination and anti-Cd58 Ab) decreased from 83.4 ± 2.98 to 51.8 ± 3.49% (*p* < 0.01, log-rank test) (Figure 9C). This trend indicates that blockade of Cd58 significantly inhibited the vaccinated immunoprotection and thus supports the notion that Cd58 plays a critical role in adaptive immunity after vaccination.

### Interaction Between Cd58 and Cd2

To evaluate the association between Cd58 and Cd2, a cellular interaction assay was initially performed on Cd58-expressing HEK293T cells by incubating the cells with FITC-Cd2. Through FCM analysis, the Cd58-expressing HEK293T cells clearly bound to the FITC-Cd2 protein in a dose-dependent manner (shown in Figure S4A in Supplementary Material). Next, a CoIP assay was conducted to provide direct evidence for the association (shown in Figure S4B in Supplementary Material). Results showed that a strong interaction exists between the Cd58 and Cd2 proteins. These observations provide insight that Cd58 and Cd2 act as reciprocal molecules in the activation of adaptive immunity.

### Functional Association of Cd58 With Cd2

**Figure 10:**
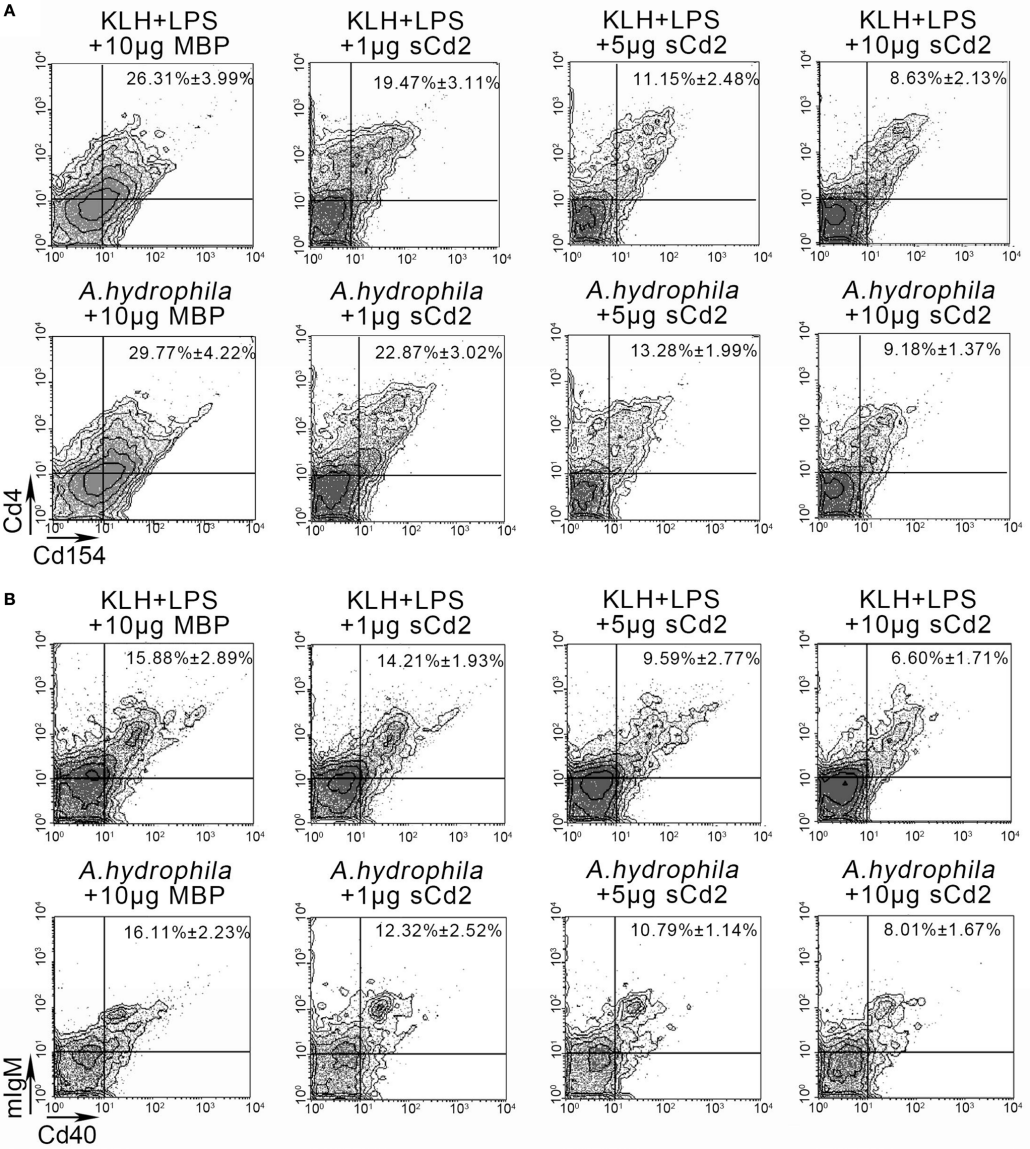
Functional evaluation of Cd58 and Cd2 interaction by introducing a recombinant soluble Cd2 protein (sCd2). **(A)**. The degree of Cd4^+^ T cell activation was represented by the percentage of Cd4^+^Cd154^+^ cells determined by flow cytometry (FCM). The experimental treatments were presented at the top of each block diagram. The data above the outlined area in each block diagram indicated the average percentage of Cd4^+^Cd154^+^ T cells in each treatment group. **(B)** The degree of B cell activation was represented by the percentage of mIgM^+^Cd40^+^ cells determined by FCM. The experimental treatments were presented at the top of each block diagram. The data above the outlined area in each block diagram indicated the average percentage of mIgM^+^Cd40^+^ B cells in each treatment group. Fishes i.p. injected with MBP were used as control. Data points were from three independent experiments (*n* = 30).

To elucidate whether Cd58 on APCs exerts its costimulatory effect through the interaction with Cd2 on Cd4^+^ T cells, a functional inhibition assay was performed by introducing the competitive binding of Cd58 with the recombinant sCd2 instead of the membrane-bound Cd2. For this procedure, the fish were administered with the sCd2 protein accompanied by immunization with different Ags (KLH plus LPS or *A. hydrophila*). As expected, the activation of Cd4^+^ T cells and mIgM^+^ B cells was significantly decreased (*p* < 0.05) as the amount of the inoculated sCd2 protein was increased from 1 to 10 µg/fish (Figures 10A,B). Specifically, the percentages of the Cd4^+^Cd154^+^ T cells in the Ag-stimulated lymphocytes decreased from 26.31 ± 3.99% (KLH and LPS plus MBP-tag control group) to 8.63 ± 2.13% (KLH and LPS plus 10 µg sCd2 group) and from 29.77 ± 4.22% (*A. hydrophila* plus MBP-tag control group) to 9.18 ± 1.37% (*A. hydrophila* plus 10 µg sCd2 group). Correspondingly, the percentages of mIgM^+^Cd40^+^ B cells in the Ag-stimulated lymphocytes decreased from 15.88 ± 2.89% (KLH and LPS plus MBP-tag control group) to 6.60 ± 1.71% (KLH and LPS plus 10 µg sCd2 group) and from 16.11 ± 2.23% (*A. hydrophila* plus MBP-tag control group) to 8.01 ± 1.67% (*A. hydrophila* plus 10 µg sCd2 group).

## Discussion

CD58 and CD2 were recognized as a pair of reciprocal immunoglobulin family members playing crucial roles in various immunological activities in humans and several other mammalian species (8, 12, 13, 17, 24, 25). Dysfunction of CD58 and CD2 in humans results in various diseases, such as multiple sclerosis, rheumatic arthritis, and psoriasis (27, 29, 30, 56). However, the occurrence and functional roles of these two molecules in non-mammalian organisms, including ancient vertebrates, such as teleost fish, remain limited. To understand CD58 and CD2 extensively, these two molecules need to be investigated in other species. In this study, we identified *cd58* and *cd2* homologs from zebrafish. A number of conserved structural lines among *cd58* and *cd2* of zebrafish and other species support the conclusion that *cd58* and *cd2* are homologous to their mammalian counterparts. The evidence includes similarity in chromosomal synteny, gene organization, and key functional domains and residues, including the extracellular IgV-like and IgC2-like domains, the key amino acids (such as Glu, Trp, and Lys residues) for CD58–CD2 interaction, the Cys residues critical for the structural integrity of the molecules, and the N-linked glycosylated sites in IgV-like and IgC2-like domains. Intriguingly, a slight difference in structural features was also seen between Cd58 and human CD58. For example, Cd58 is 119 amino acids longer in the cytoplasmic tail than the human CD58, which was encoded by two additional exons (exons 7 and 8) in the *cd58* gene. This phenomenon was also predicted to exist in several other non-mammalian species, such as reptiles and birds (e.g., 41 amino acids longer in *Gekko japonicus*, 121 amino acids longer in *Struthio camelus australis*, 112 amino acids longer in *Taeniopygia guttata*, and 56 amino acids longer in *Parus major*). Moreover, the intracellular tail of the CD58 proteins tends to be shorter from fish to mammals. The finding implies that the signal transduction function of CD58 itself has been gradually weakened along with vertebrate evolution, and thus, the functional role of CD58 may undergo a change from a direct executor for signaling transduction into a major regulator for intercellular adhesion. Actually, the existence of a GPI-anchored form of CD58 in humans may support this notion. In this case, the CD58 protein was anchored outside the cellular membrane by a total lack of the transmembrane and intracellular domains, which is beneficial for CD58 to bind its reciprocal molecules more flexibly out of the cell (3, 11). In mammals, once CD58 is bound, the CD2 membrane protein in T cells triggers cellular activation and IL-2 production by interacting with its downstream adaptor proteins (e.g., CD2BP1, CD2BP2, and CD2AP1) *via* the PPLPRPR motif in the intracellular tail (57). Unlike the change in CD58, the intracellular tail of CD2 was found to be well conserved from fish to mammals throughout the vertebrate evolution. These observations suggest that CD58 may have a higher divergence and a special evolutionary history in comparison with CD2. In accordance with this notion, several different forms of CD58 were found in some species (e.g., soluble form in sheep), and an overall lower identity of sequence alignment was seen between porcine CD58 and sheep CD58 compared with that of humans (12). In addition, the CD58 and CD2 genes are located on the same chromosome and are structurally related in humans; however, CD58 was commonly absent in rodents, and there even exists a hypothesis that CD58 might arise from CD2 by a gene duplication event after the split of mouse and humans (14, 58). Our study showed that CD58 and CD2 had occurred individually in zebrafish, suggesting the origin of these two molecules as early as in teleost fish. Although the CD58 and CD2 genes were located on different chromosomes in fish, these two molecules are similar in amino acid sequences (above 33%) and functional domain structures. These findings implied that the CD58 and CD2 genes may have evolved from a common ancestor by gene duplication, which originated from primitive vertebrates. The separate chromosomal distribution of *cd58* and *cd2* genes in zebrafish implies the existence of a split event of CD58/CD2 gene complex in fish lineage, which might be resulted from the gene insertion and transposition events that frequently occurred during the whole genome duplication in early vertebrate evolution. This phenomenon is similar to the genomic organization of many other fish genes, such as CD28 and CTLA4 or MHC class I and class II genes, which are closely linked in humans and almost all other vertebrates but teleosts (59, 60). To fully understand this issue, further investigation is needed to clarify the synteny organization for *cd2* gene. This depends on an improved zebrafish genome database, in which more complete information needed for the annotation of immediate neighbor genes around the *cd2* locus becomes available.

Functionally, the regulatory roles of CD58 and CD2 interactions mainly focused on cellular immunity, such as CD8^+^ T and NK-mediated cytokine production and cytolysis, as well as apoptosis of activated peripheral T cells (2, 24, 25). For example, a substantial proportion of CD8^+^ T cells in adults of humans lack the expression of the CD28 molecule. This CD28^−^CD8^+^ T cell subset was characterized by potent effector functions but impaired responses to antigenic challenge. As CD28 is a primary T-cell costimulatory receptor, in most cases, the CD80/86–CD28 axis plays a crucial role in the initiation of T-cell activation. Thus, the alternative costimulatory pathway contributing to the activation of CD28^−^CD8^+^ T cells remains to be clarified. In a recent study, engagement of the CD2 molecule by its ligand CD58 dramatically activated the proliferation, cytokine production, and effector function in CD28-deficient T subset. This finding indicates that CD58–CD2 interaction is a primary costimulatory pathway for human CD8^+^ T cells that lack CD28 (25). In addition, the existence and expansion of adaptive NK-cell subsets were closely associated with the infection of viruses, such as human cytomegalovirus (HCMV). A majority of adaptive NK cells were found to express the activating receptor NKG2C and CD57. Recently, CD2 and CD58 were found to be greatly upregulated on the adaptive NK cells and fibroblasts under HCMV infection. Blockade of CD2 and CD58 resulted in diminished production of IFN-γ and TNF-α by adaptive NK cells in response to HCMV-infected cells. This finding indicates that CD58–CD2 interactions are pivotal for the activation and function of adaptive NK cells in human HCMV infection (24). However, whether the CD58–CD2 interaction is essential for other adaptive immune activities, such as for CD4^+^ Th-initiated humoral immunity, remain poorly understood. Moreover, due to the absence of CD58 in mouse and other rodent models, previous understanding for CD58 and CD2 comes largely from humans and depends on *in vitro* systems. *In vivo* functional characterization of CD58 and CD2 remains limited and somewhat enigmatic. For example, mice bearing a CD2 knockout only exhibit very partial defects in immune responses, although CD2 plays crucial roles in the human system (8, 61). Therefore, alternative animal models are needed to improve the current knowledge on the functional performance of CD58 and CD2 *in vivo*, which would also benefit in depicting the evolutionary history of the CD58 and CD2 families.

Recently, zebrafish has become a powerful model system for immunology with its versatility and high degree of conservation in innate and adaptive immunities (26, 62–64). In this study, we found that CD58–CD2 interaction provided a primary costimulatory signal for the full activation of adaptive humoral immunity by using zebrafish model, thus uncovering a new functional mechanism of CD58/CD2 underlying host immunity. A number of experimental evidence supports this proposal. For example, Cd58 and Cd2 were distributed on Mhc-ii^+^ APCs and Cd4^+^ Th cells, and these two molecules could be significantly upregulated upon antigen stimulation. This finding provided preliminary insights that Cd58 and Cd2 were closely associated with the costimulatory functions between APCs and Cd4^+^ Th cells in antigen-elicited adaptive humoral immunity. By knockdown and blockade of Cd58 or Cd2, the activation of antigen-specific Cd4^+^ Th cells was significantly impaired, mIgM^+^ B cell activation and Ab (IgM) production were inhibited, and defense against bacterial infections post-vaccination was diminished. Notably, *in vivo* administration of fish with a sCd2 also significantly reduced the antigen-stimulated activation of Cd4^+^ Th cells and B cells. It provided functional evaluation for the association of Cd2 with Cd58, in which the soluble Cd2 may competitively inhibit the interaction of natural Cd2 with Cd58 between Cd4^+^ T cells and APCs. This observation was confirmed by the direct binding of soluble Cd2 to Cd58 expressed on HEK293T cells as determined through FCM and CoIP assays. This result suggests that the sCd2 with extracellular domains may be used as a negative regulator to suppress the hyperimmune reactions induced by CD58, which might have potential application for therapeutic purpose. Naturally, a soluble CD58 variant produced by alternative splicing was identified from humans. It plays an inhibitory role in the CD58/CD2 costimulatory pathway (13, 65). However, whether a natural soluble CD2 exists in cells remains unclear. Thus, further identification of this variant is needed to clarify this issue.

In conclusion, this study revealed that CD58 and CD2 interactions provide a primary costimulatory signal for the full activation of CD4^+^ Th-mediated adaptive humoral immunity in zebrafish, adding a new costimulatory signaling pathway to the regulatory network of adaptive immunity. This finding makes zebrafish an attractive model organism for understanding CD58/CD2-mediated immunity and diseases. The occurrence of CD58 and CD2 in zebrafish also suggests that these two costimulatory signals may have originated as early as in teleost fish, which would be beneficial in mapping the evolutionary history of the CD58 and CD2 families throughout the vertebrate evolution.

## Ethics Statement

All animal work in this paper was conducted according to relevant national and international guidelines. All animal care and experimental procedures were approved by the Committee on Animal Care and Use and the Committee on the Ethic of Animal Experiments of Zhejiang University.

## Author Contributions

Conceived and designed the experiments: TS and J-zS. Performed the experiments: TS and WS. Analyzed the data: TS, WS, J-yZ, X-xX, A-fL, and L-xX. Contributed reagents/materials/analysis tools: L-xX and J-zS. Wrote the manuscript: TS, X-xX, and J-zS.

## Conflict of Interest Statement

The authors declare that the research was conducted in the absence of any commercial or financial relationships that could be construed as a potential conflict of interest.

## Abbreviations

LFA-3, lymphocyte function-associated antigen-3; GPI, glycosylphosphatidylinositol; mAbs, monoclonal antibodies; FCM, flow cytometry; HEK293T, human embryonic kidney 293 cell transformed by expression of the large T antigen from SV40; UTR, untranslated region; IgV-like, Ig variable region-like; IgC2-like, Ig constant region-like; i.p, intraperitoneal injected; Mhc-ii, MHC class II; FITC-Cd2, FITC-conjugated recombinant soluble Cd2 protein; siRNA, small interfering RNA; shRNA, short hairpin RNA; LV, lentivirus; Cd4^+^ T_KLH_, KLH-stimulated Cd4^+^ T cell; Cd4^+^ T*_A.h_, A. hydrophila*-stimulated Cd4^+^ T cell; *lck*, tyrosine-protein kinase; OVA, ovalbumin; KLH, keyhole limpet hemocyanin; LPS, lipopolysaccharide; PBLs, peripheral blood leukocytes; HKLs, head kidney lymphocytes.
