# Supplementary material for: Costimulatory Function of Cd58/Cd2 Interaction in Adaptive Humoral Immunity in a Zebrafish Model
Source: Front Immunol. 2018 May 31;9:1204. doi: 10.3389/fimmu.2018.01204 (PMC5990624; doi:10.3389/fimmu.2018.01204)
Supplement: Supplementary file 1 [file data_sheet_1.doc]

Supplementary Material

# Costimulatory function of Cd58/Cd2 interaction in adaptive humoral immunity in a zebrafish model

**Tong Shao^1^, Wei Shi^1^, Jia-yu Zheng^1^, Xiao-xiao Xu^1^, Ai-fu Lin^1^, Li-xin Xiang^*1^, Jian-zhong Shao^*1,2^**

**Correspondence:** Jianzhong Shao and Lixin Xiang, College of Life Sciences, Zhejiang University, Hangzhou 310058, China, Tel.: +86 (571) 8820 6582; Fax: +86 (571) 8820 6582. Email: [shaojz@zju.edu.cn](mailto:shaojz@zju.edu.cn); xianglx@zju.edu.cn

SUPPLEMENTAL TABLE S1. Primers used in the experiments

| Primer Name  /Accession Number | Sequence (5’ to 3’) | Application |
| --- | --- | --- |
| *cd58*-F1 | GGAGGCTCGTCTTCATAC | ORF cloning |
| *cd58*-R1 | TAAGTTATTTCTGCTGTCGT | ORF cloning |
| *cd2*-F1 | TTCCTTCTGCCGATTGAGAAAA | ORF cloning |
| *cd2*-R1 | CACGCAGGCATCTCATTCCATT | ORF cloning |
| *cd58*- F2 | CGCGGATCCGCCACCATGGGAAGAAGACTC | Eukaryotic expression |
| *cd58*- R2 | CCGGAATTCGTCTCCATCCTCTGCATTAGT | Eukaryotic expression |
| *cd58*- F3 | CCGCTCGAGGCCACCATGGGAAGAAGACTCTGCGGA | Eukaryotic expression |
| *cd58*- R3 | CCGGAATTCGGTCTCCATCCTCTGCATTAGT | Eukaryotic expression |
| *cd2*-F2 | CCGCTCGAGGCCACCATGAGCTGCCAAAAC | Eukaryotic expression |
| *cd2*-R2 | CCGGAATTCGTTCCATTGGACGGTAG | Eukaryotic expression |
| *cd2*-F3 | GGGTACCGATGAGCTGCCAAAACAATC | Prokaryotic expression |
| *cd2*-R3 | CAAGCTTGGCATCAAATCCAAAAAGTG | Prokaryotic expression |
| *cd58*-F5 | TACAGATGGACCACAGCACAAT | Real-time RT-PCR |
| *cd58*-R5 | CAGCGATCATTACAATCAAAGC | Real-time RT-PCR |
| *cd58*-F6 | GTGTCAAGAGCAAGCCTTCGTT | Real-time RT-PCR |
| *cd58*-R6 | GTCGCTTCTTCAGTGGTTTCAGT | Real-time RT-PCR |
|  |  |  |
| *cd2*-F4 | AAGACGGCACGAAGAAGC | Real-time RT-PCR |
| *cd*2-R4 | GAGGCTGAAACTTTGTCACTTA | Real-time RT-PCR |
| *cd154-*F | CGAATGGCAACAGGGCACAAGAATG | Real-time RT-PCR |
| *cd154-*R | TCTAAACACTCCTGCTGATGATGCC | Real-time RT-PCR |
| *lck-*F | AGATGAATGGTGTGACCAGTGTA | Real-time RT-PCR |
| *lck*-R | GATCCTGTAGTGCTTGATGATGT | Real-time RT-PCR |
| *mhc-iia-*F | CTCTCATTGAACTATGGATC | Real-time RT-PCR |
| *mhc-iia*-R | CAAAGCTGTCAGTTGCAGTTG | Real-time RT-PCR |
| il-4/13a -F | GCACTGTATTCGTCTCGGGTTTTA | Real-time RT-PCR |
| il-4/13a -R | TTTTCCCCAGATCTACAAGGAAGA | Real-time RT-PCR |
| il-4/13b-F | CTGTTGGTACTTACATTGGTCCCC | Real-time RT-PCR |
| il-4/13b-R | AGTGTCCTGTCTCATATATGTCAGGT | Real-time RT-PCR |
| *Il-2-*F | CGCACACACTGATGATGATGAGGAT | Real-time RT-PCR |
| *Il-2-*R | TTCTGCCTCCATTCGTTCATCCA | Real-time RT-PCR |
| *cd58*-siRNA-F1 | GATCCCCTCGCTGTGATTATCTGCATTTCAAGAGAATGCAGATAATCACAGCGATTTTTA | RNAi |
| *cd58*-siRNA-R1 | AGCTTAAAAATCGCTGTGATTATCTGCATTCTCTTGAAATGCAGATAATCACAGCGAGGG | RNAi |
| *cd58*-siRNA-F2 | GATCCCCCAGCGGAAAGCTGCAAATGTTCAAGAGACATTTGCAGCTTTCCGCTGTTTTTA | RNAi |
| *cd58*-siRNA-R2 | AGCTTAAAAACAGCGGAAAGCTGCAAATGTCTCTTGAACATTTGCAGCTTTCCGCTGGGG | RNAi |
| *cd58*-siRNA-F3 | GATCCCCCAGCCGGACTCTGTTTATATTCAAGAGATATAAACAGAGTCCGGCTGTTTTTA | RNAi |
| *cd58*-siRNA-R3 | AGCTTAAAAACAGCCGGACTCTGTTTATATCTCTTGAATATAAACAGAGTCCGGCTGGGG | RNAi |
| Scrambled siRNA  -sense | GATCCCCAGTAGCTTACGATAGAACGTTCA  AGAGACGTTCTATCGTAAGCTACTTTTTTA | RNAi |
| Scrambled siRNA  -antisense | AGCTTAAAAACGTTCTATCGTAAGCTACTT  CTCTTGAAAGTAGCTTACGATAGAACGGGG | RNAi |
| β-actin –F | ACACCTTCTACAATGAGCTG | Gene expression |
| β-actin -R | CTGCTTGCTGATCCACATCT | Gene expression |

F: Forward primer;

R: Reverse primer.

**SUPPLEMENTAL FIGURE S1**


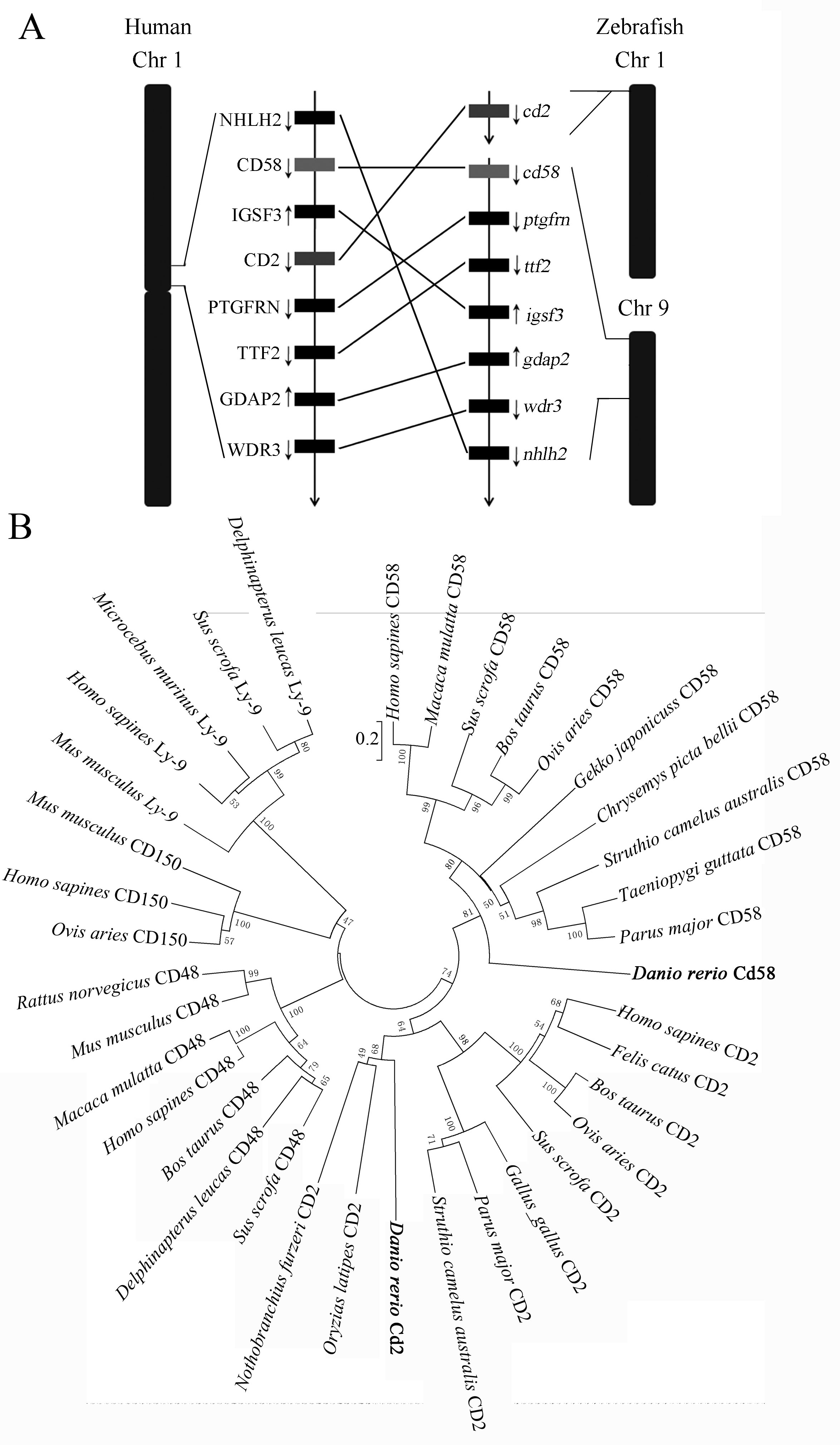


**SUPPLEMENTAL FIGURE S1** **A,** comparative analysis of the chromosomal location of CD58 and CD2 genes between zebrafish and humans. Arrows indicate gene orientation. **B,** phylogenetic analysis of CD58 and CD2. Phylogenetic tree shows the relationship of Cd58 and Cd2 in zebrafish and other species. The unrooted phylogenetic tree is constructed via the neighbor-joining method based on the amino acid alignment (CLUTAL W) of amino acid sequences. CD48, CD150 and Ly-9 which have the closest genetic relationship to the CD58 and CD2, were included. The accession numbers of mammalian sequences included in the phylogenetic analyses are as follows: *Homo sapiens* CD58, NM_001779.2; *Macaca mulatta* CD58, NM_001267785.1; *Sus scrofa* CD58, NM_213795.1; *Ovis aries* CD58, XM_015092195.1; *Bos Taurus* CD58, XM_010803137.1; *Chrysemys picta bellii* CD58, XM_008173651.1; *Gekko japonicas* CD58, XM_015409986.1; *Struthio camelus australis* CD58, XM_009689671.1; *Parus major* CD58, XM_015644770.1; *Taeniopygia guttata* CD58, XM_012569957.1. *Homo sapiens* CD2, NM_001328609.1; *Sus scrofa* CD2, NM_213776.1; *Bos Taurus* CD2, NM_001011676.3; *Struthio camelus australis* CD2, XM_009682712.1; *Parus major* CD2, XM_015648339.1; *Ovis aries* CD2, XM_012179323.2; *Felis catus* CD2, NM_001009841.1; *Gallus gallus* CD2, XM_015298201.1; *Oryzias latipes* CD2, XM_004069513.2; *Nothobranchius furzeri* CD2, XM_015952350.1. *Homo sapiens* CD48, NM_001778.3; *Macaca mulatta* CD48, XM_015113619.1; *Mus musculus* CD48, NM_007649.4; *Rattus norvegicus* CD48, NM_139103.1; *Sus scrofa* CD48, NM_001243714.1; *Bos Taurus* CD48, NM_001046002.1. *Delphinapterus leucas* CD48, XP_022415234.1; *Homo sapiens* CD150, NP_001317683.1; *Mus musculus* CD150, NP_038758.2; *Ovis aries* CD150, NP_001035378.1; *Mus musculus* Ly-9, BAE96310.1; *Homo sapiens* Ly-9, AAH27920.1; *Sus scrofa* Ly-9, XP_013852312.1; *Delphinapterus leucas* Ly-9, XP_022415384.1; *Microcebus murinus* Ly-9, XP_012604652.1.

**SUPPLEMENTAL FIGURE S2**


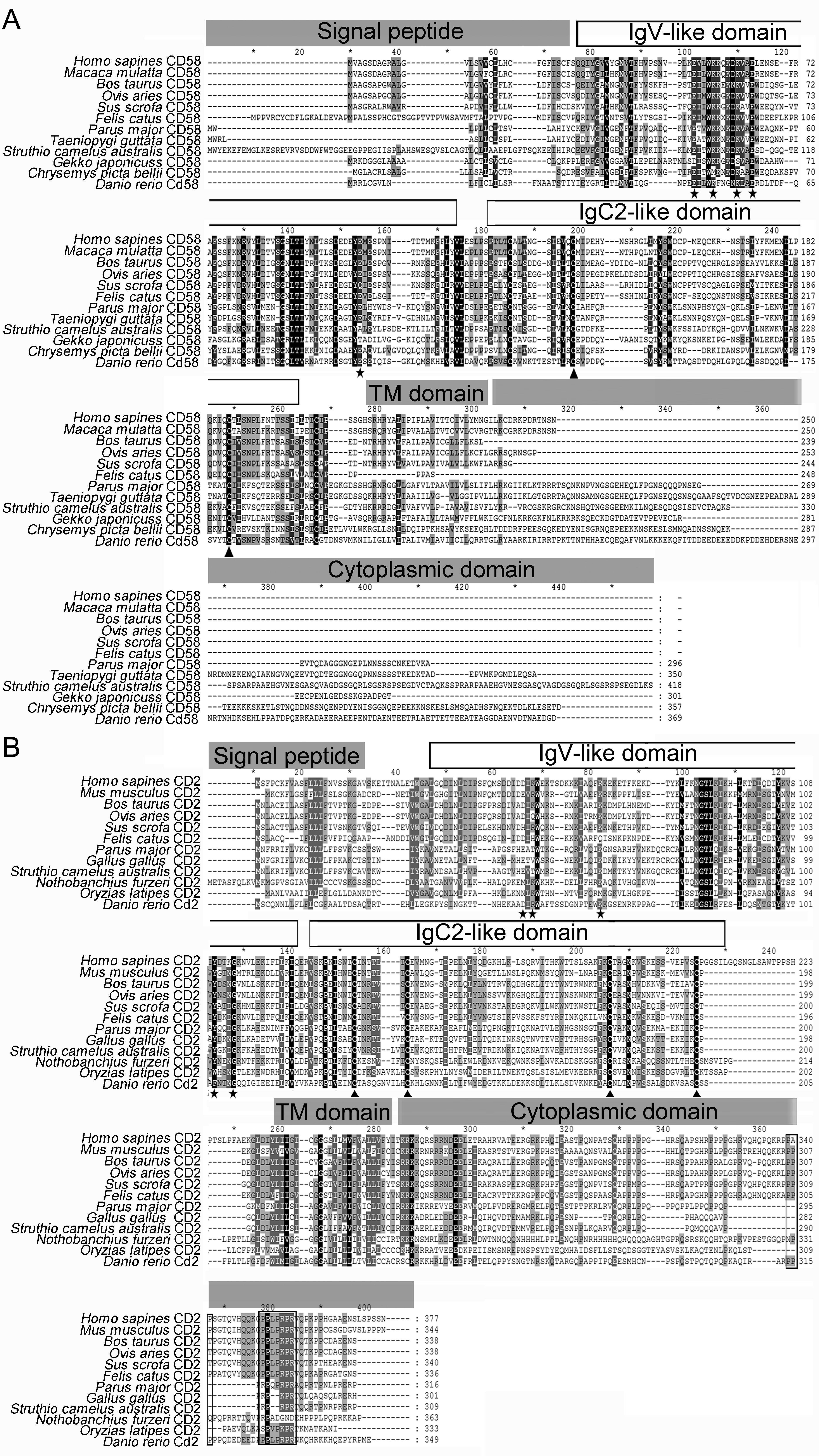


**SUPPLEMENTAL FIGURE S2** Multiple alignment analysis of CD58 **(A)** and CD2 **(B)** molecules. Residues shaded in black are completely conserved across all species aligned, and residues shaded in gray are similar in terms of side chains. The dashes in the amino acid sequences indicate gaps introduced to maximize alignment. “▲” and “★” below the alignment indicate the conserved cysteine and the key amino acid residues (Glu46, Trp49, Lys50, Lys55, Glu58 and Glu99 for Cd58, Asp49, Arg51, Lys65, Phe103 and Gly107 for Cd2, all of which are involved in the interaction of CD58 and CD2), respectively. The conserved IgV- and IgC2-like domains are indicated above the alignment.

**SUPPLEMENTAL FIGURE S3**


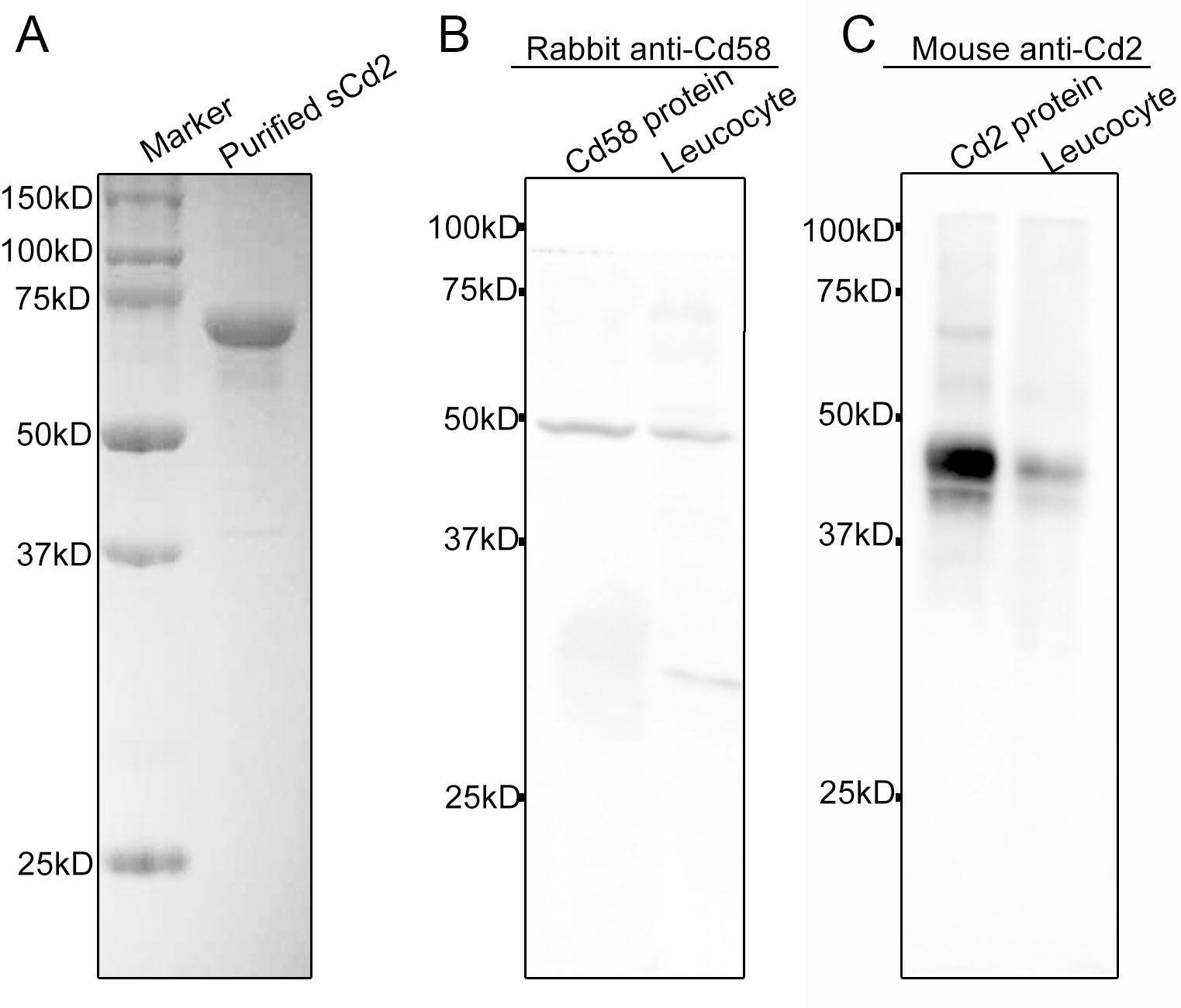


**SUPPLEMENTAL FIGURE S3**. Assays for the specificity of anti-Cd58 and anti-Cd2 Abs. **(A)** SDS–PAGE analysis of recombinant Cd2-MBP protein. The standard molecular weight protein markers are at the right side of the panel. (**B–C**) Western blot analyses showing the effectiveness of the purified rabbit anti-Cd58 and mouse anti-Cd2 Abs that specifically bind to the corresponding recombinant target proteins expressed in eucaryotic systems and proteins from leukocytes.

**SUPPLEMENTAL FIGURE S4.**


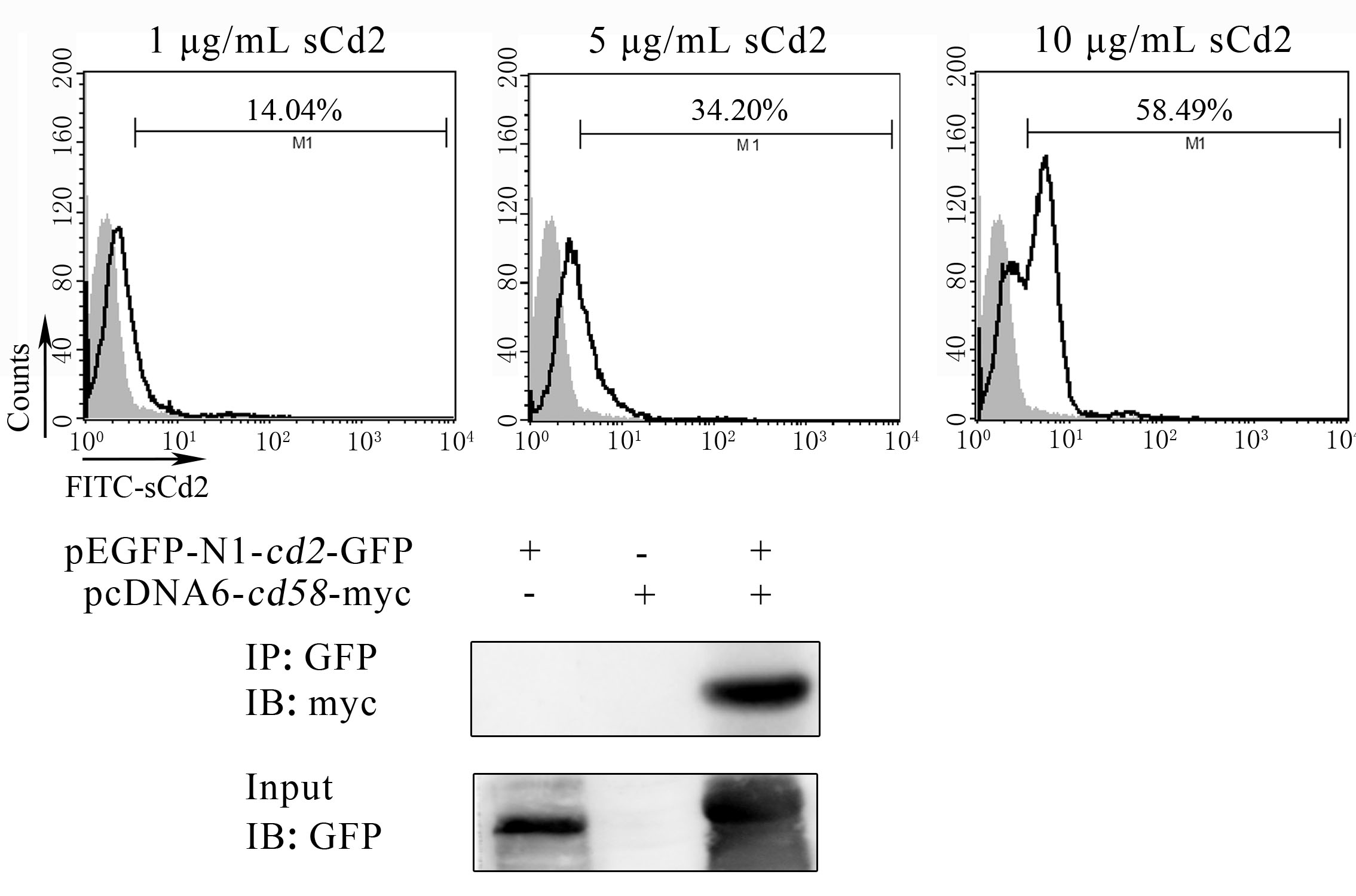


**SUPPLEMENTAL FIGURE S4.** Assays for the association of Cd58 and Cd2 molecules. **(A)** Detection of the association via FCM with FITC-conjugated recombinant Cd2 (FITC-sCd2) and Cd58 proteins expressed on HEK293T cells. Nontransfected HEK293T cells with no sCd2 protein were devised as negative controls (black curve). **(B)** Detection of the association via co-immunoprecipitation (Co-IP) assay. HEK293T cells were co-transfected with pcDNA6-myc-*cd58* fused with myc tag and pEGFP-*cd2* fused with GFP tag, followed by immunoprecipitation with GFP tag-Ab and detected by Myc/GFP tag-Abs.
